# The antibacterial activity of a photoactivatable diarylacetylene against Gram-positive bacteria

**DOI:** 10.3389/fmicb.2023.1243818

**Published:** 2023-09-22

**Authors:** Ryan Waite, Candace T. Adams, David R. Chisholm, C. H. Cole Sims, Joshua G. Hughes, Eva Dias, Emily A. White, Kathryn Welsby, Stanley W. Botchway, Andrew Whiting, Gary J. Sharples, Carrie A. Ambler

**Affiliations:** ^1^Department of Biosciences, Durham University, Science Site, Durham, United Kingdom; ^2^LightOx Limited, Newcastle, United Kingdom; ^3^Department of Physics, Durham University, Science Site, Durham, United Kingdom; ^4^Central Laser Facility, Research Complex at Harwell, Science and Technology Facilities Council, Rutherford Appleton Laboratory, Harwell, United Kingdom; ^5^Department of Chemistry, Durham University, Durham, United Kingdom

**Keywords:** antimicrobial resistance, photodynamic therapy, Gram-positive bacteria, lipopolysaccharides, reactive oxygen species

## Abstract

The emergence of antibiotic resistance is a growing threat to human health, and therefore, alternatives to existing compounds are urgently needed. In this context, a novel fluorescent photoactivatable diarylacetylene has been identified and characterised for its antibacterial activity, which preferentially eliminates Gram-positive over Gram-negative bacteria. Experiments confirmed that the Gram-negative lipopolysaccharide-rich outer surface is responsible for tolerance, as strains with reduced outer membrane integrity showed increased susceptibility. Additionally, bacteria deficient in oxidative damage repair pathways also displayed enhanced sensitivity, confirming that reactive oxygen species production is the mechanism of antibacterial activity. This new diarylacetylene shows promise as an antibacterial agent against Gram-positive bacteria that can be activated *in situ*, potentially for the treatment of skin infections.

## Introduction

The discovery of penicillin in the 1920s marked the beginning of the golden age of antimicrobial discovery, which ended in the 1980s when the last new major class was discovered (Hutchings and AWT, 2019). This era also saw widespread antibiotic use, and overuse, providing selective pressure for acquisition and dissemination of antimicrobial resistance (AMR) across various species, resulting in a global health problem often heralded as the ‘silent pandemic’. In 2019, AMR was responsible for around 1.3 million deaths globally (Murray et al., 2022). Additionally, there is clear evidence that bacteria are becoming increasingly resistant to existing antibiotics, making the discovery of new therapeutic approaches imperative. Post-1980 efforts to discover new antibiotics have largely focussed on small molecule discovery as single drugs and in combination (Terreni et al., 2021). However, with the rate at which resistance is developing, additional therapeutic approaches are required.

Light-activated, cytotoxic compounds, where light exposure is required to trigger ‘cell killing’ capabilities, have advantages in minimising systemic, off-target toxicity in humans. Photoactivated cytotoxic activity, often via the generation of reactive oxygen species (ROS), has been widely employed to disrupt the viability of prokaryotes in addition to eukaryotes; for example, Methylene Blue (Wainwright, 1996; Wainwright et al., 2017). A key advantage of this modality is that ROS are indiscriminate in their cellular targets including lipids, proteins and nucleic acids, making it unlikely for bacterial resistance to develop (Maisch, 2015). However, the major roadblock to the use of many photoactivated antimicrobials in a therapeutic context is the photosensitisers themselves; most are high molecular weight porphyrin structures that are generally insoluble, exhibit inadequate pharmacokinetic properties, cannot permeate Gram-negative bacteria, or display limited selectivity between prokaryotic and eukaryotic cells (Yin and Hamblin, 2015).

We recently characterised a new class of low molecular weight donor-acceptor diphenylacetylene photosensitisers that exhibit cytotoxic activity towards eukaryotes when activated by UV, violet or corresponding two-photon near-IR (infrared) irradiation (Gala De Pablo et al., 2018, 2020; Chisholm et al., 2019, 2020; Hughes et al., 2022). These compounds operate ostensibly by generating ROS upon photoactivation, leading to organelle damage and extensive membrane disruption that causes cell death. Compared to many photosensitisers utilised for the elimination of bacteria, these diphenylacetylenes exhibit a significantly lower molecular weight (350–500 Da) and more ‘drug-like’ structure, making them attractive candidates for photoactivated antimicrobial approaches. Accordingly, we designed and subsequently screened, a number of derivatives for their ability to penetrate certain bacteria, and after photoexcitation, then cause bacterial damage. Herein, we report the first example of a promising diarylphenylacetylene Compound **2**, a photosensitiser with antibacterial action against Gram-positive bacteria.

## Materials and methods

### Compound 2 absorption and emission spectra

The structures of 6 diarylacetylenes candidates that were screened for biological function are shown in Supplementary Figure S1. Details of the chemical synthesis of Compound **2** can also be found in the Supplementary material. Absorption spectra were obtained using a Perkin Elmer Cary 60 spectrometer and emission spectra using an Agilent Cary Eclipse spectrometer. For absorption spectra, 5 μM solutions of Compound **2** in CHCl_3_ and DMSO were added to 10 mm path length quartz optical cuvettes (Hellma) and absorbances recorded at 1 nm intervals. Extinction coefficient measurements were determined in triplicate using measurements obtained from absorption readings at the respective λ_max_ exhibited by Compound **2** in each solvent at concentrations from 5 to 30 μM. Extinction coefficient values are expressed as an average of the three replicates, with the standard deviation. Emission spectra were obtained at 1 nm intervals from 100 nM solutions in quartz cuvettes, as specified above, using excitation at λ = 380 nm, and normalised according to the respective maximal intensity values.

### Quantum yield measurement

The quantum yield from one-photon excitation was determined using LightOx17 (Quantum yield 0.67) in Toluene as standard. Compound **2** was measured at varying concentration in each of the solvents aiming for absorbances of 0.1 and below, these corresponded to concentrations in the 0.50 – 2 μM range. Absorbances of each compound in solution were recorded between 300 and 1,000 nm and the corresponding fluorescence intensity was measured between 250 and 700 nm.

Quantum yield was calculated using the relative method. The absorbance and fluorescence of compounds in solution were measured at multiple concentrations and compared to a reference via the following equation (Würth et al., 2013).


$$\phi_{S}=\phi_{R}\frac{Grad_{S}}{Grad_{R}}{\left(\frac{n_{s}}{n_{R}}\right)}^{2}$$

where Grad is the gradient obtained by plotting the integrated fluorescence, I , against the absorbance, $1-10^{-A}\text{,}$ and n is the refractive index of the solvents. The absorbance was measured at 390 nm using a Cary 60 UV–Vis, aiming for absorbances 0.1 and below. The fluorescence was measured using the same excitation wavelength (390 nm) by a Cary Eclipse Fluorescence Spectrophotometer and integrated between 400 and 700 nm. LightOx17 in toluene was used as the reference for all calculations (Wainwright et al., 2017). For each compound between 5 and 7 concentrations were measured with 3 repeats.

### Bacteria

*Bacillus subtilis* 168 (ATCC 23857), *Staphylococcus epidermidis* (ATCC 12228), *Pseudomonas fluorescens* (ATCC 13525) and *Escherichia coli* FDA strain Seattle 1946 (ATCC 25922) were obtained from the American Type Culture Collection. The *E. coli* K-12 wild-type BW25113 [*rrnB3* Δ*lacZ4787 hsdR514* Δ(*araBAD*)*567* Δ(*rhaBAD*)*568 rph-1*] is the parent strain for the Keio collection (Baba et al., 2006) and was used as the wild-type strain for comparisons with insertion–deletion derivatives: JW3596 (Δ*rfaC*::*kan*), JW2669 (Δ*recA*::*kan*), JW0097 (Δ*mutT*::*kan*), JW3879 (Δ*sodA*::*kan*), JW1648 (Δ*sodB*::*kan*), JW1638 (Δ*sodC*::*kan*), JW0598 (Δ*ahpC*::*kan*), JW3914 (Δ*katG*::*kan*) and JW2663 (Δ*gshA*::*kan*).

### Photoactivated bacterial growth inhibition

All bacteria were cultivated in LB (Miller) broth in an orbital shaker (VWR) at 30–37°C. Overnight cultures were prepared by inoculating a single, isolated colony into 10 mL of LB broth followed by incubation with shaking for 16–20 h. Bacteria exposed to photoactivatable compound were placed in a LightOx PhotoReact 365 Lightbox (Merck) and exposed to light at a wavelength of 365 nm for 5 min, with an energy intensity of 13 mW/cm^2^ (total energy delivered: 3.9 J/cm^2^). For assays requiring half of a 96-well or agar plate to be irradiated, a section of black card was used to mask the relevant samples.

For bacterial overlays, 50 mL of 1.5% LB agar was poured into a 100 × 100 × 20 mm square petri dish (Sarstedt) and once solidified, 15 mL of 0.75% LB soft agar inoculated with 200 μL of bacteria from an overnight culture was poured onto the surface. Serially diluted concentrations of compound were applied to the overlay in 6 μL volumes. Plates to be photoactivated were then exposed to light at 365 nm and incubated at 30°C for 24 h before imaging in a Bio-Rad Gel Doc XR+ System.

For growth curves, 5 mL of LB broth in a 15 mL Falcon tube (Sarstedt) was inoculated with 50 μL of bacteria from an overnight culture. Compound **2** was added to give a final concentration of 2 μM and incubated in the dark with shaking at 30°C for 30 min. Samples (100 μL) were pipetted into the wells of a 96-well plate, with 8 repeats per sample. Half of the plate was covered, and the rest irradiated at 365 nm. Growth was monitored at OD_600nm_ every 5 min for 24 h in a plate reader (Biotek Synergy HT) and data normalised against the negative control containing media alone.

### Viability assays

A semi-microcuvette (Sarstedt) containing 2 mL of LB broth inoculated with 50 μL of bacteria from an overnight culture was incubated with shaking at 30°C until early log phase, OD_600nm_ 0.2. Samples of 1 mL were transferred to a 24-well plate and incubated with Compound **2** at 2 μM for 30 min at 30°C. Serial dilutions (10-fold) were performed, 30 μL sample in 270 μL LB, in a 96-well plate. Samples in the 24-well plate were irradiated, incubated for 15 min at room temperature and 30 μL samples removed and serial 10-fold dilutions made as before. Samples (10 μL) were applied to an LB agar plate in triplicate and incubated for 16–20 h. Colonies at appropriate dilutions were enumerated and viability determined in colony forming units (CFU) per ml.

### Monitoring loss of membrane integrity using propidium iodide

Bacteria were cultivated as in viability assays and 500 μL of the culture transferred to a 1.5 mL microcentrifuge tube containing Compound **2** to give a final concentration of 2 μM. A control sample with 500 μL of the culture and 0.2% DMSO was set up in parallel. Samples were incubated at 30°C for 30 min and cells pelleted by centrifugation at 17,000 g for 4 min. Cell pellets were resuspended in 200 μL 1× PBS containing 7.5 μM propidium iodide (PI; ThermoFisher). 3 × 50 μL volumes of each sample were applied to a 96-well plate, transferred to a Biotek Synergy HT and fluorescence measurements made every 2 min for 20 min with excitation at 485 nm and emission at 645 nm. PBS (50 μL) was then added to sample wells and 50 μL 100% ethanol added to additional samples as a positive control for loss of membrane integrity and cell killing. The plate was placed in the PhotoReact 365 lightbox, irradiated for 5 min, returned immediately to the plate reader and fluorescence monitored every 2 min for 1 h.

### Confocal microscopy

For confocal microscopy, cell pellets were prepared as in the propidium iodide assays with 500 μL of the culture transferred to a 1.5 mL microcentrifuge tube containing Compound **2** to give a final concentration of 2 μM. Samples were incubated at 30°C for 30 min and cells pelleted by centrifugation at 17,000 g for 4 min, then resuspended in 200 μL of Baclight solution containing 10 μM SYTO9 and 60 μM PI. A 10 μL sample was applied to a 1 cm x 1 cm 1.5% agarose (Bioline) pad on a microscope slide with a cover slip placed on top. The slide was imaged using a 63× lens on a confocal microscope (Zeiss 800 Airyscan) with Compound **2** being imaged with the airyscan function using a 405 nm laser and an emission filter of 450–550 nm, SYTO 9 using a 488 nm laser and 550–580 nm filter and PI using a 488 nm laser and an emission filter of 600–650 nm. Samples were irradiated using the 405 nm laser at 30% power for 1 min (total energy: 90 mJ/cm^2^) on the microscope. A time lapse video was assembled from images taken every 6 s for 10 min.

## Results

### Photosensitiser screening for antibacterial activity

**Figure 1:**
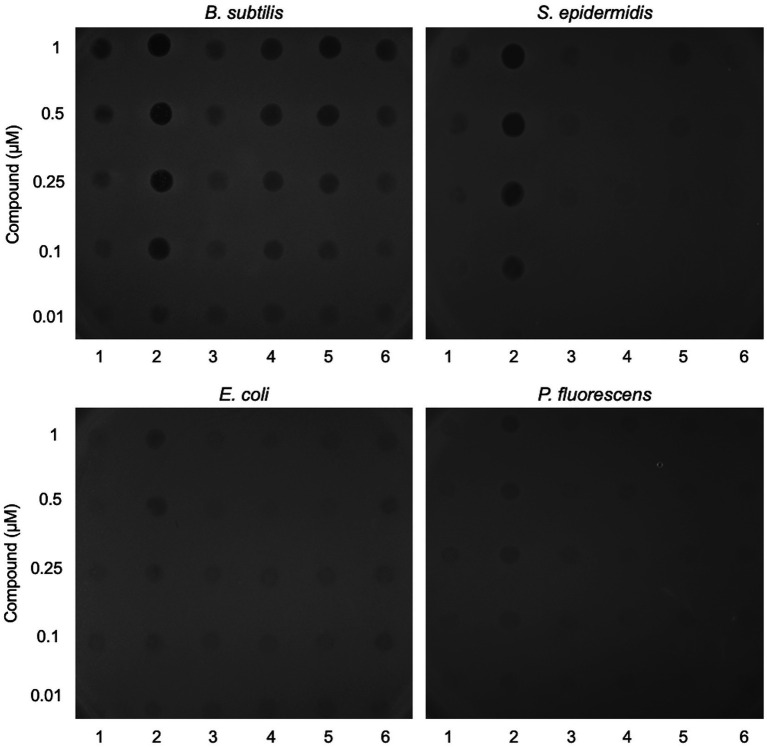
Screening lead compounds for antibacterial activity against Gram-positive and Gram-negative bacteria. Two-fold dilutions of six lead compounds (labelled 1–6) were applied in 6 μL volumes to the surface of a soft agar overlay inoculated with *Bacillus subtilis*, *Staphylococcus epidermidis*, *Escherichia coli* or *Pseudomonas fluorescens*. The LB agar plates were exposed to light at 365 nm for 5 min and then incubated for 24 h at 37°C prior to imaging. Controls without light activation or with application of DMSO are shown in Supplementary Figure S2.

**Figure 2:**
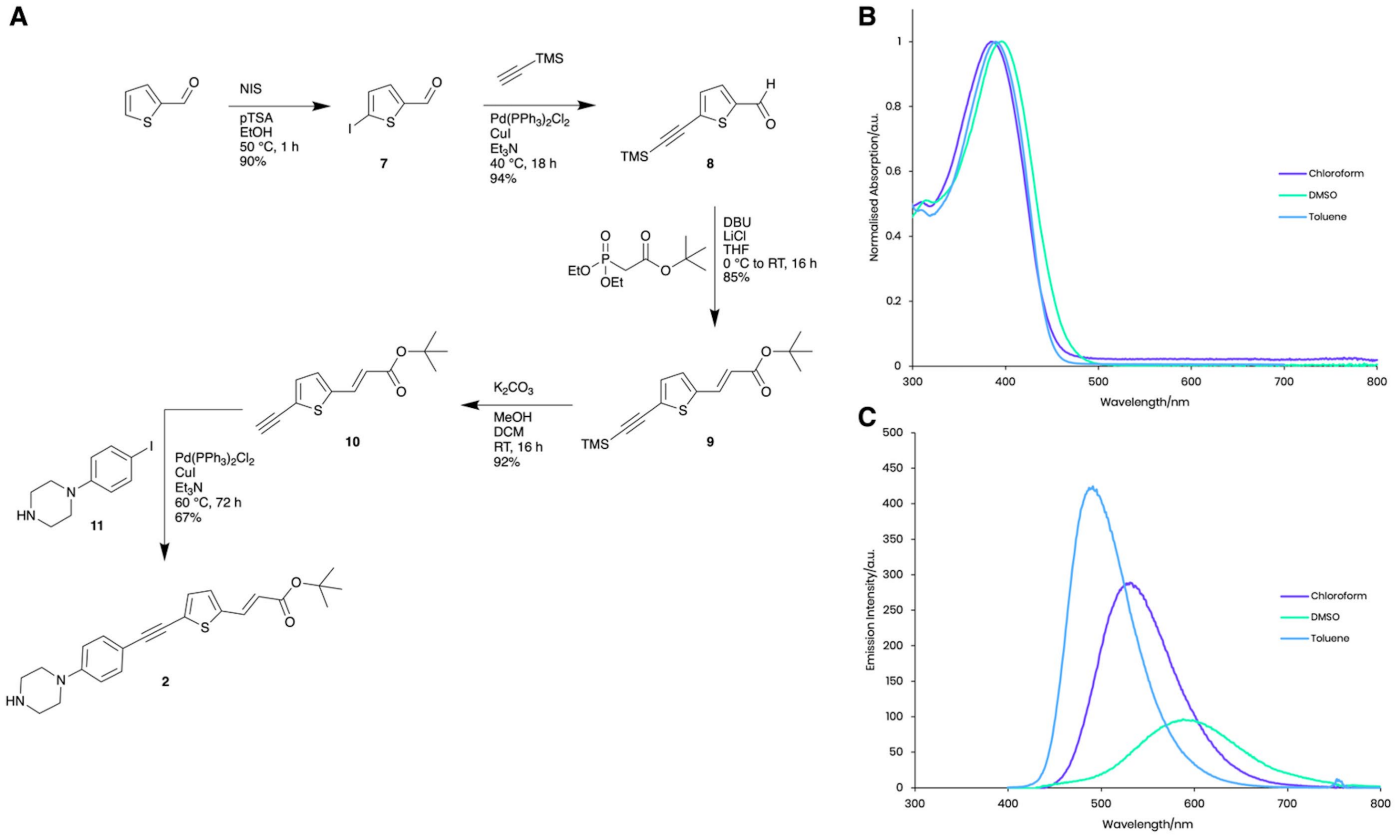
Photophysical properties of Compound **2**. **(A)** Synthesis and structure of Compound **2**. **(B)** Normalised absorption spectra of Compound **2** in chloroform, DMSO and toluene. **(C)** Emission spectra of Compound **2** in chloroform, DMSO and toluene with excitation at the respective absorption peak maxima.

Six candidate diarylacetylene photosensitisers were screened for antibacterial activity against two Gram-negative (*Escherichia coli* and *Pseudomonas fluorescens*) and two Gram-positive species (*Bacillus subtilis* and *Staphylococcus epidermidis*). The compounds were applied to the surface of an overlay containing each bacterial species and were subsequently activated by exposure to light at 365 nm, followed by incubation for 24 h (Figure 1). Unirradiated samples were set up in parallel (Supplementary Figure S2; Supplementary material) and none of the tested compounds affected the growth of any of the four bacterial species under these conditions (Supplementary Figure S2). In contrast, samples activated by exposure to near-UV light showed distinct zones of growth inhibition (Figure 1). Most of the compounds tested restricted bacterial growth at the highest concentrations, with *B. subtilis* proving most sensitive. Compound **2** displayed the greatest efficacy, notably with the two Gram-positives, *B. subtilis* and *S. epidermidis* (Figure 1). Compound **2** was therefore selected for further study; its synthesis is detailed in Figure 2 and Electronic Supplementary material section 2.

### Photophysical properties of Compound 2

**Figure 3:**
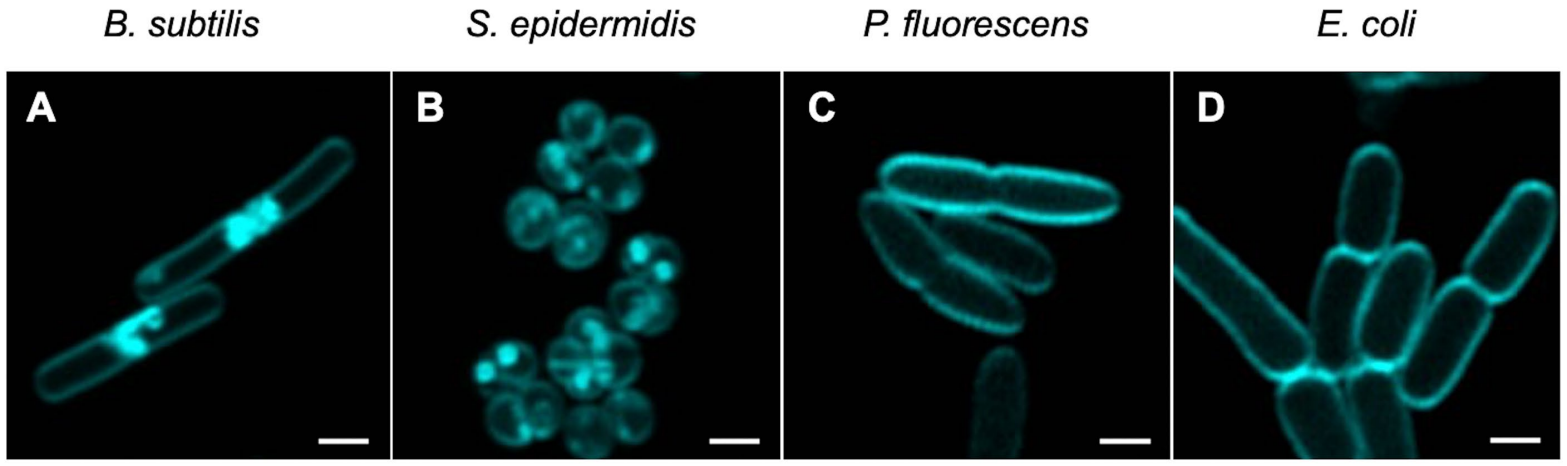
Visualisation of non-activated Compound **2** in bacterial cells. Mid-log phase bacteria were incubated with 2 μM of Compound **2** for 30 min before centrifugation and resuspension in 1x PBS. Resuspended cells were applied to a 1% agarose pad and imaged using confocal microscopy with cyan false colour imaging of the compound using a 405 nm laser with detection at 450–550 nm using the Airyscan function. The bar represents 1 μm.

Compound **2** exhibits similar solvatochromatic absorption (Figure 2B) and emission (Figure 2C) behaviour in toluene, chloroform and ethanol as other donor-acceptor structures (Chisholm et al., 2019, 2020). In nonpolar solvents, high intensity, shorter wavelength fluorescence emission (toluene, l_max_ = 491 nm, Φ = 0.8 and chloroform l_max_ = 531 nm, Φ = 0.50) was observed, while much weaker emission with a significant bathochromic shift in the considerably more polar solvent, DMSO (l_max_ = 588 nm, Φ = 0.001) was shown, presumably due to aggregation and self-quenching effects. These photophysical properties enabled us to visualise the intracellular localisation of Compound **2** using fluorescence microscopy techniques (Figure 3).

### Effect of Compound 2 on bacterial growth

**Figure 4:**
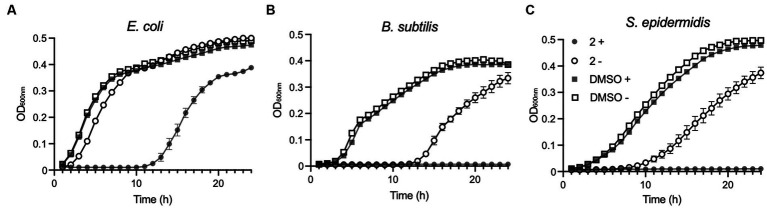
Effect of Compound **2** on bacterial growth. *Escherichia coli* **(A)**, *B. subtilis* **(B)** and *S. epidermidis* **(C)** were cultivated in LB broth at 37°C in 96-well plates in a plate reader with continuous shaking. Growth was monitored at OD_600nm_ in samples exposed to light at 365 nm for 5 min (filled symbols) or without light treatment (open symbols). Samples contained 2 μM Compound **2** or 0.2% DMSO indicated by circles or squares, respectively.

Compound **2** was next examined for its ability to inhibit the growth of *E. coli, B. subtilis,* and *S. epidermidis* in liquid cultures. Unfortunately, *P. fluorescens*, as an obligate aerobe, could not be tested due to the restricted oxygen availability in microtitre plates. Bacterial growth in response to treatment with 2 μM Compound **2** was followed by optical density measurements over a 24-h period with half of the samples irradiated with 365 nm light at the outset alongside unirradiated controls (Figure 4).

2 μM was selected as the concentration due to the difference in susceptibility displayed in liquid cultures and on agar, with bacterial lawns being shown to be more susceptible than the corresponding species in a liquid growth assay. Therefore, 2 μM was selected as this was the lowest concentration of Compound **2** that would completely inhibit the growth of *B. subtilis* in LB broth following photoactivation (Figure 4).

*E. coli* exposed to the light-activated Compound **2** experienced a substantial (10 h) lag in growth relative to an unirradiated control, although there was a resumption in growth beyond this time point (Figure 4A). Unirradiated *E. coli* grown in the presence of the compound did show a modest delay in growth but quickly recovered thereafter (Figure 4A). The two Gram-positive species, *B. subtilis* and *S. epidermidis,* showed much greater susceptibility to Compound **2** exposure (Figures 4B,C). In light-exposed samples, growth ceased immediately and did not resume over the 24-h monitoring period. Growth inhibition was also evident in unirradiated cultures exposed to Compound **2** and indicates some, albeit reduced, toxicity in the absence of light activation in these species at this relatively high treatment concentration. No differences in growth were detected with appropriate vehicle in the presence or absence of light, demonstrating that the compound is solely responsible for bacterial growth inhibition in these species.

### Effect of Compound 2 on bacterial viability

**Figure 5:**
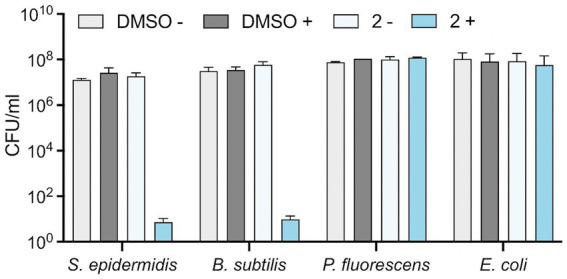
Effect of Compound **2** on bacterial viability. Bacteria at mid-log phase of growth (30 μL) were transferred to a clear 24-well plate were mixed with 270 μL LB broth. Serial (10-fold) dilutions were performed and 10 μL of each dilution was applied to the surface of an LB agar plate in the presence of 2 μM Compound **2** or 0.2% DMSO. The plate was then activated by irradiation at 365 nm and samples diluted and spotted again. Both irradiated and non-irradiated plates were incubated for 24 h before enumeration of colonies to determine CFU/ml. Data are the mean and standard error of three independent experiments.

To determine whether Compound **2** exhibits bacteriostatic or bactericidal properties at this test concentration, we performed a viability assay. The four bacterial species were grown to early-log phase prior to addition of 2 μM Compound **2** and exposure to 365 nm light. Appropriate controls without irradiation and equivalent concentrations of DMSO were conducted in parallel. Serial dilutions of the bacteria were applied to the surface of agar plates and CFU/ml calculated (Figure 5). No reduction in viability of any of the bacterial species was observed in the controls, with or without light, or samples incubated with Compound **2** but without irradiation (Figure 5). Similar results were obtained with the two Gram-negative species, *E. coli and P. fluorescens*, consistent with resistance towards the light-activated effect of the compound (Figure 5). *S. epidermidis* and *B. subtilis*, in contrast, showed a dramatic reduction in viability after light exposure, both showing a 6-log reduction in survival compared to the non-irradiated controls (Figure 5).

**Figure 6:**
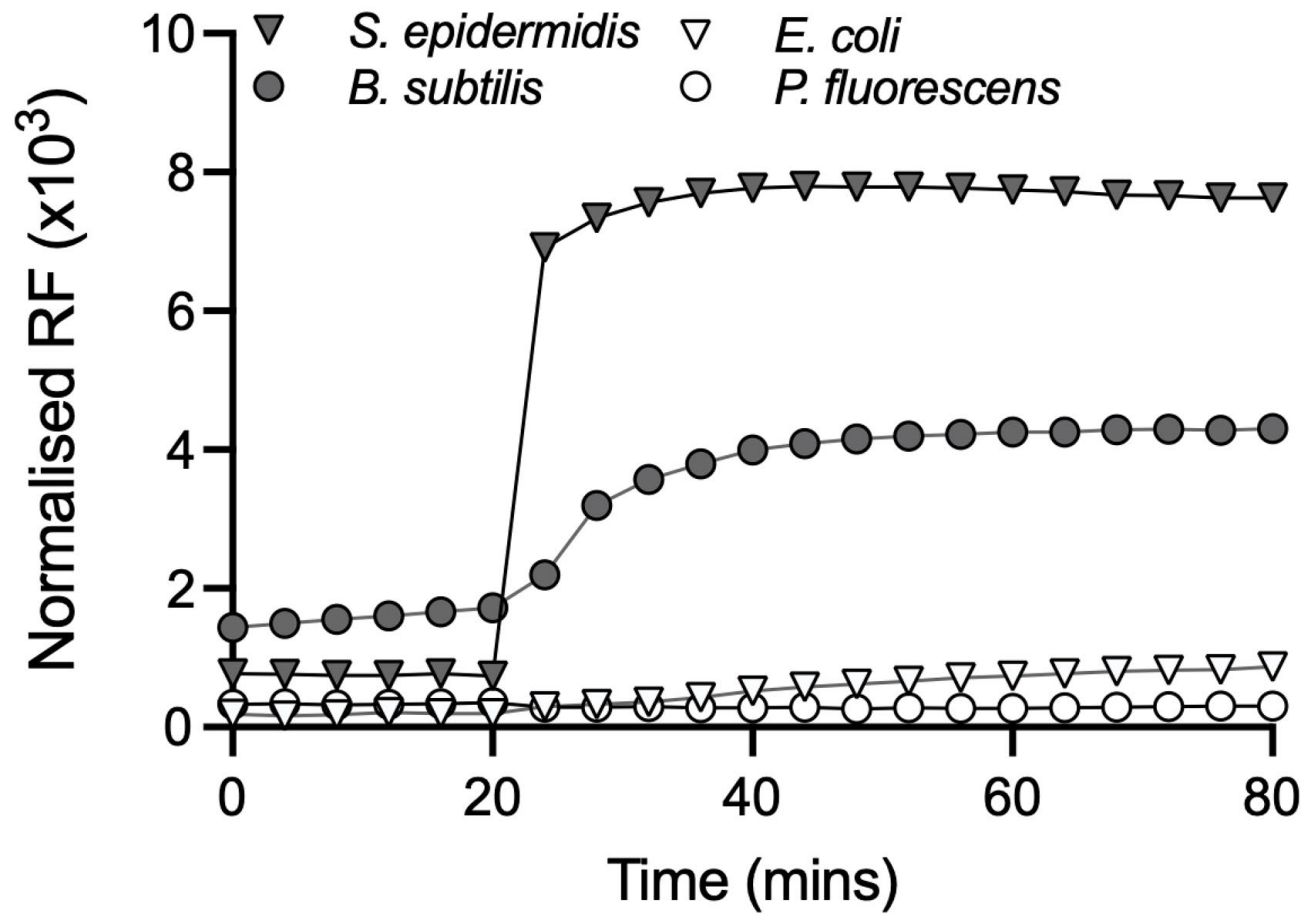
Effect of Compound **2** on bacterial membrane integrity. Bacteria were grown to mid-log phase in the presence or absence of 2 μM Compound **2** as described in the Material and Methods. The relative fluorescence units (RFU × 10^3^) at an emission of 645 nm were normalised against controls containing appropriate control concentrations of DMSO. All samples were exposed to light at 365 nm after 20 min and incubation continued for another 60 min. An ethanol positive control for membrane damage using ethanol was conducted in parallel but is not included in the graph.

To explore this further, propidium iodide (PI) was used to assess loss of membrane integrity in cells exposed to Compound **2**. PI is a membrane-impermeable dye that fluoresces in the presence of chromosomal DNA only when it can penetrate the cell envelope and thus serves as a reporter for severe membrane damage, and, with some caveats, for cell death (Netuschil et al., 2014). The four bacterial species were incubated in the presence of 2 μM Compound **2** and then exposed to 365 nm light after 20 min. PI fluorescence was monitored throughout the experiment from 0 to 80 min. *S. epidermidis* and *B. subtilis* show a small increase in PI fluorescence prior to irradiation, indicating that there may be a slight loss of membrane integrity, especially with *B. subtilis* (Figure 6). A significant increase in PI fluorescence was evident with both Gram-positive species following photoactivation (Figure 6), consistent with rapid loss of cell viability (Figure 5). *E. coli* and *P. fluorescens* treated with the same concentration (2 μM) of Compound **2** only showed a minor increase in PI fluorescence after light activation. Ethanol controls were conducted in parallel and are shown in Supplementary Figure S3.

### Real-time monitoring of bacterial membrane integrity

To provide further evidence that membrane disruption by Compound **2** is dependent on photoactivation, a real time BacLight assay was employed. The assay utilises SYTO 9, a membrane permeable dye that fluoresces when bound to chromosomal DNA, while PI, as mentioned above, only enters cells when membrane integrity is severely compromised and displaces SYTO 9 due to its higher affinity for DNA (Stocks, 2004). Bacteria were grown to early-log phase, treated with 2 μM Compound **2** in the presence of the two dyes and visualised by microscopy. Compound **2** was activated by light at 405 nm and images were captured over 10 min to monitor changes in fluorescence.

**Figure 7:**
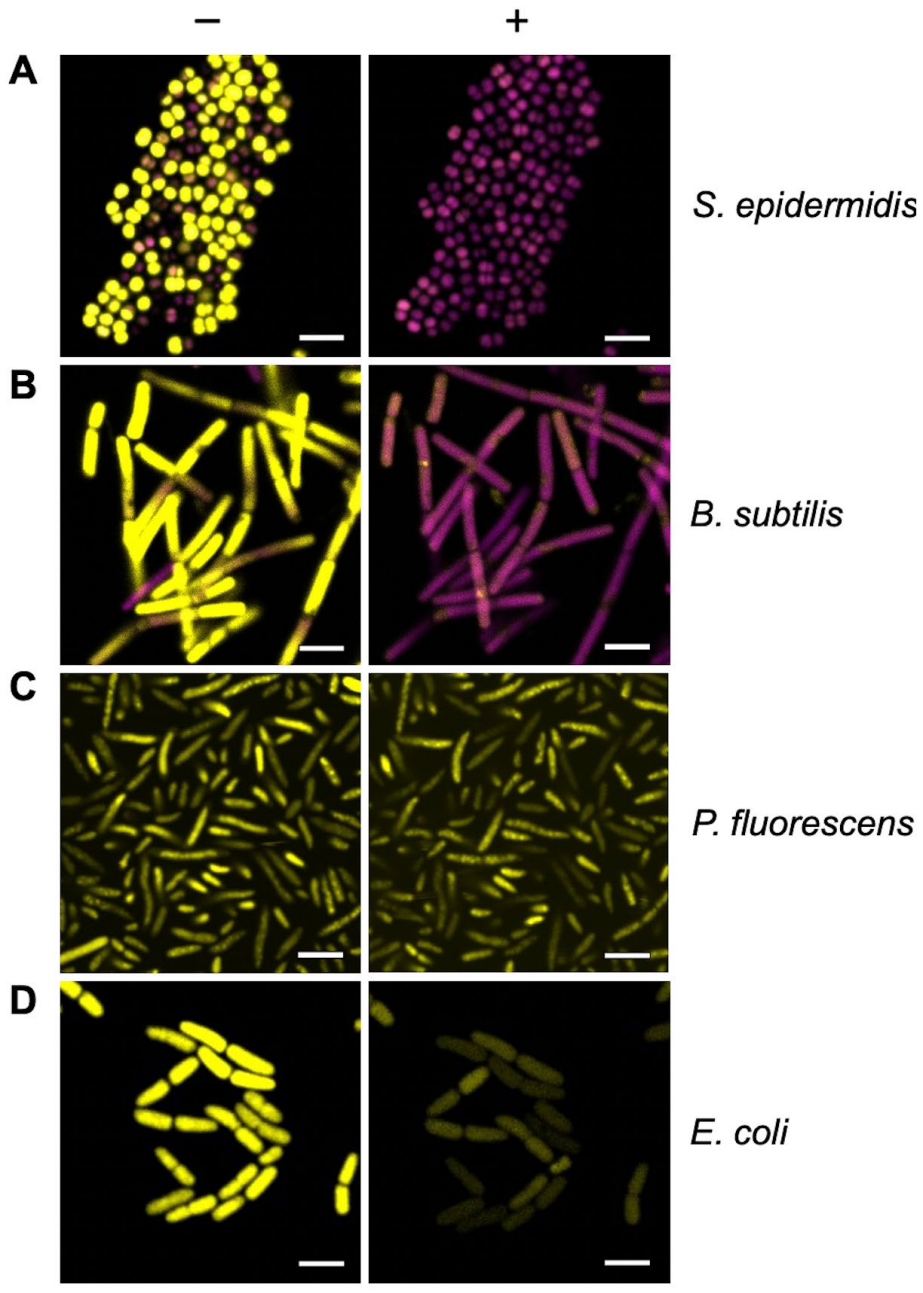
Real-time monitoring of bacterial membrane integrity. The BacLight assay of membrane integrity following photoactivation of Compound **2**. *S. epidermidis* **(A)**, *B. subtilis* **(B)**, *P. fluorescens* **(C)** and *E. coli* **(D)** in mid-log phase of growth were stained with PI (magenta) and SYTO 9 (yellow) and imaged by confocal microscopy without light activation (−) or 10 min after photoactivation with the 405 nm laser (+). The laser was applied at 30% power for 1 min. The bar represents 3 μm.

Most of the *B. subtilis* and *S. epidermidis* bacteria were stained with SYTO 9 prior to light activation (Figures 7A,B; SYTO 9 coloured yellow) indicating these cells possessed intact cell envelopes and were alive. Photoactivation resulted in a rapid fluorescence change, with all cells stained with PI after 10 min (Figures 7A,B; Supplementary Video S1; PI coloured magenta). These changes indicate significant membrane damage, and likely cell death, due to the activated Compound **2**, allowing the influx and subsequent fluorescence from PI. In contrast, *E. coli* and *P. fluorescens* with cells exposed to Compound **2** retained the SYTO 9 dye after light activation with no indication of membrane disruption (Figures 7C,D).

### Visualisation of Compound 2 in bacterial cells

Utilising the fluorescence properties of Compound **2**, we next determined if the compound could be detected intracellularly in the four bacterial species under investigation. Bacteria were grown to mid-log phase, as before, and visualised by microscopy. Some association of the compound with the cell surface was evident with both Gram-positive species, although there were also dense patches evident, either as (potential) aggregates at the surface or accumulation within the cytosol (Figures 3A,B). Clustering of Compound **2** at the poles of *B. subtilis* cells was particularly apparent (Figure 3A), notably between cells undergoing division potentially because these areas are more accessible. These concentrated patches were entirely absent in the two Gram-negative species, where Compound **2** appeared to associate solely with the cell surface (Figures 3C,D).

### The Gram-negative outer membrane protects against Compound 2 toxicity

Gram-negative bacteria are resistant to many antibiotics due to the impermeability of their lipopolysaccharide-rich (LPS) outer membrane to hydrophobic molecules (Zgurskaya and Rybenkov, 2020). The experiments performed so far suggested that the tolerance of Compound **2** by *E. coli* and *P. fluorescens* arises from a similar mechanism and that entry to the cytosol is a requirement for toxicity. To investigate this in more detail we examined the susceptibility of three *E. coli* strains that differ in their lipopolysaccharide composition. The *E. coli* strain (ATCC 25922; O^+^) used in Figures 1, 3, 4 has a ‘smooth’ phenotype due to the presence of typical O-antigens decorating the core lipid A (Liu et al., 2019). The K12 laboratory strain of *E. coli* (CGSC 7636) lacks O-antigen polysaccharides (O^−^) and is therefore slightly more permeable to hydrophobic compounds (Delcour, 2009). Finally, an *E. coli* K12 strain carrying a deletion of *rfaC* (*waaC*) is unable to complete addition of core sugars during lipid A assembly and is therefore highly susceptible to penetration by antibiotics and disinfectants as a result (Pagnout et al., 2019).

**Figure 8:**
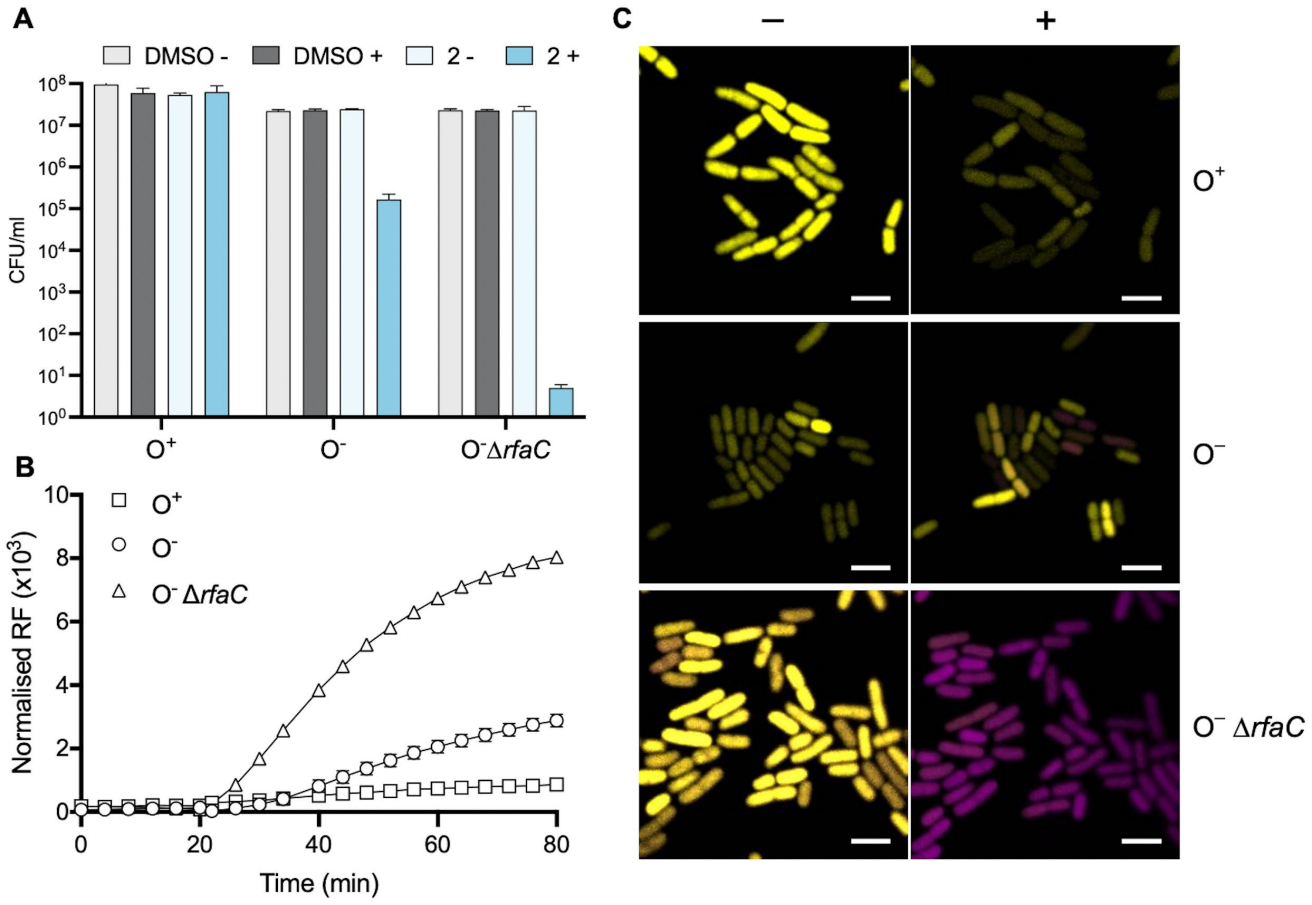
Role of the *E. coli* outer membrane in protecting against Compound **2** toxicity. **(A)** The viability of *E. coli* O^+^, O^−^ and Δ*rfaC* cells was evaluated as in Figure 5 with exposure to 2 μM Compound **2** with or without light activation. **(B)** PI assay of *E. coli* strains to monitor loss of membrane integrity. **(C)** Real-time imaging of *E. coli* strains stained with PI (magenta) and SYTO 9 (yellow) by confocal microscopy. Images taken prior to light exposure (−) and 10 min after photoactivation (+) as indicated. The bar represents 3 μm.

These three *E. coli* strains were examined using several techniques employed above to examine the importance of the outer membrane in Gram-negative tolerance of Compound **2**. Viability was assessed following exposure to the compound with or without light activation (Figure 8A). The results show that the O^+^ strain is relatively tolerant of the compound even when activated (Figure 8A) in accordance with earlier results (Figure 5). In contrast, the O^−^ K12 strain showed a 2-log decrease in viability relative to the non-irradiated control (Figure 8A). This increase in susceptibility was even more evident with the Δ*rfaC* mutant with a 6-log reduction in viability with the light-activated Compound **2** (Figure 8A).

These *E. coli* strains were also probed for their ability to internalise PI in response to photoactivation of Compound **2** (Figure 8B). As before (Figure 6), the *E. coli* O^+^ strain showed only a slight increase in fluorescence, consistent with a lack of damage to the outer membrane (Figure 8B). Upon light exposure, increased PI fluorescence was much more apparent with the O^−^ strain and considerably more so with the *rfaC* mutant (Figure 8B). These results suggest that damage to the outer membrane allows entry of Compound **2** and that subsequent photoactivation of the compound leads to severe damage to membrane integrity facilitating PI uptake. The BacLight assay was employed to visualise any real-time change in membrane integrity in individual *E. coli* cells (Figure 8C). There was little change in *E. coli* O^+^ cells in response to light activation of the compound, as noted earlier (Figure 7D), although a few cells showed some reduction in SYTO 9 intensity (Figure 8C). However, several cells show an increase in the presence of PI fluorescence after photoactivation in the O^−^ strain (Figure 8C). In the Δ*rfaC* strain all the cells have taken up PI (Figure 8C) following photoactivation of Compound **2**. Collectively, these experiments establish that the outer membrane of Gram-negative bacteria is responsible for protection against Compound **2** toxicity.

### Susceptibility of *E. coli* strains deficient in oxidative damage and tolerance pathways

**Figure 9:**
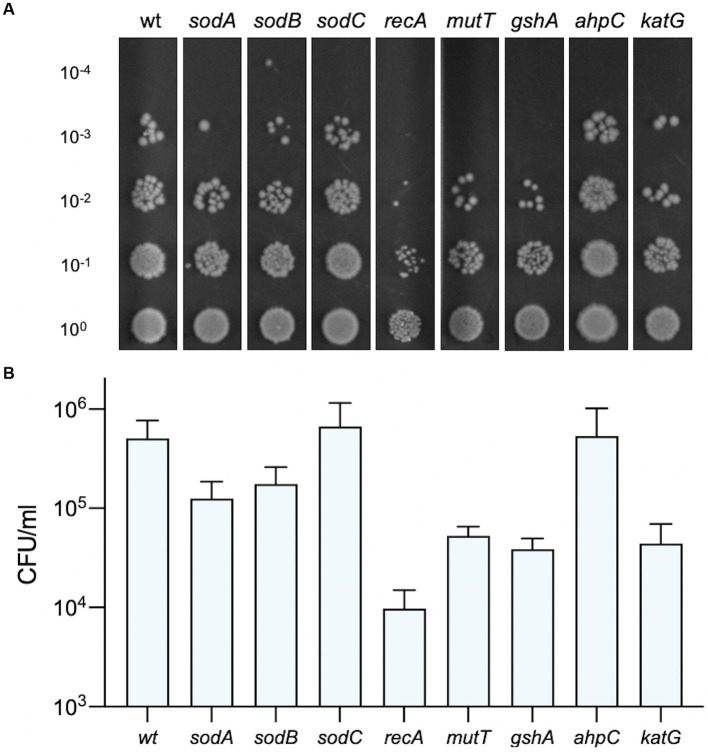
Susceptibility of *E. coli* strains deficient in oxidative damage and tolerance pathways. The viability of bacteria exposed to 2 μM Compound **2** after photoactivation is shown. Serial dilutions of bacteria were applied to LB agar plates and colonies counted to determine CFU/ml. A representative image of each strain is shown **(A)** and the CFU/ml **(B)** represents the mean and standard error of three independent experiments.

Photosensitisers are known to elicit the generation of reactive oxygen species (ROS) when activated by light and these radicals can cause considerable damage to cellular lipids, proteins and DNA (Baptista et al., 2017). It is possible that intracellular photoactivation of Compound **2** is responsible for the rapid loss of membrane integrity and viability observed with *B. subtilis* and *S. epidermidis* (Figures 5–7). Since *E. coli* K12 strains showed some elevated susceptibility to photoactivated Compound **2** because of the absence of O antigens, we utilised Keio collection deletion mutants defective in ROS detoxifying and repair pathways to investigate their importance in compound tolerance. The wt *E. coli* BW25113 showed moderate susceptibility to photoactivated Compound **2** at 2 μM in a viability assay (Figure 9) and similar levels of tolerance in strains lacking the SodC [Zn-Cu] superoxide dismutase and AhpC, an alkyl hydroperoxide reductase (Figure 9). SodC is localised to the periplasm (Benov et al., 1995) and may suggest that any ROS damage by compound photoactivation is limited to the cytosol. AhpC, in complex with AhpF (Kamariah et al., 2018), detoxifies hydrogen peroxide and organic hydroperoxides and the lack of increased susceptibility in a Δ*ahpC* strain could indicate that peroxides are not a major product of Compound **2** activation. The other mutants tested (*sodA*, *sodB*, *recA*, *mutT*, *gshA* and *katG*) showed increased sensitivity (10–100-fold) to light-activated Compound **2**, giving strong evidence that ROS are generated and exert damaging effects on survival (Figure 9). The susceptibility of the two cytosolic superoxide dismutases (SodA and SodB) implicate superoxide production as a feature of compound activation. KatG (catalase) works in concert with these dismutases as it eliminates hydrogen peroxide produced by these manganese and iron-dependent SODs (Imlay, 2013). GshA is required for the biosynthesis of the antioxidant glutathione and the knockout strain is more sensitive to a range of oxygen radicals (Łyżeń et al., 2022). MutT hydolyzes 8-oxo-dGTP to 8-oxo-dGMP to remove it from the nucleotide pool and prevent its misincorporation into DNA (Ito et al., 2005) RecA is required for homologous recombination and is necessary for double-stranded DNA break repair arising from single and double-strand breaks generated by oxygen radicals (Horii et al., 1980). All these mutants showed an increased susceptibility to photoactivated Compound **2**, with the Δ*recA* mutant being notable in showing a 100-fold decrease in viability relative to the wild type and confirming that chromosomal DNA also sustains damage. Taken together, these results indicate that significant ROS generation in the bacterial cytosol takes place in response to photoactivation of Compound **2**.

## Discussion

Photosensitisers have been characterised as an effective antimicrobial treatment for resistant bacteria. In this study, Compound **2**, a novel diarylacetylene, exhibited bactericidal properties against the Gram-positive bacterial species in response to photoactivation. The excellent activity of Compound **2** may be due to two unique structural characteristics. First, the basic phenylpiperazine moiety of the donor region will likely be protonated under the bacterial culture conditions. This would aid localisation to the net negatively charged membrane structures (Gottenbos et al., 2001) of most bacteria and, thus, potentially aid internalisation. Second, the thiophene moiety of the acceptor region may aid the generation of suitably reactive excited states during photoactivation by promoting intersystem crossing (Fonseca et al., 2006) and thus potentially increase/modulate the elicitation of ROS generation. Photosensitisers are classified as Type I-IV based on the chemical mechanisms required to illicit oxidative stress and photocytotoxicity. These four types are further grouped into direct and indirect photosensitisers. The most common are indirect, oxygen-dependent, photosensitisers, Type I and II. These generate ROS through either an electron transfer reaction with oxygen (Type I), or through an energy transfer to form singlet oxygen (Type II). Direct, oxygen-independent, photosensitisers, Type III and IV, directly react with biologically substrates and free radicals to cause photocytotoxicity. Type III PSs interact via a triplet-doublet reaction, while Type IV PSs require photoisomerisation to enable target binding (Scherer et al., 2017; Yao et al., 2021). In an antimicrobial context, both Type I and II are photoexcited to generate large amounts of ROS that disrupt bacterial membranes causing cell death (Das et al., 2017). Photoactivation of Compound **2** leads to both membrane and intracellular damage, indicating it might be a Type I photosensitiser. There is some indication that internalisation of Compound **2** is required for bactericidal action. In Gram-positives, the uptake of the nucleic acid stain PI is rapid following activation, suggesting a rapid loss of envelope integrity. In contrast, minimal uptake of PI occurs in Gram-negatives unless the outer membrane permeability is compromised, as seen in *E. coli* O^−^ and O^−^ Δ*rfaC* strains (Figure 8).

Reactive oxygen species are a grouped class of oxygen containing molecules that include superoxide anion, hydroxyl radical, hydrogen peroxide, singlet oxygen, and peroxyl radicals. Analysis of Compound **2** function in *E. coli* mutant strains lacking ROS repair and detoxification pathways suggests that superoxide is in part responsible for cellular damage and death. Deletion of either of the two SOD enzymes located in the cytoplasm show greater susceptibility than the one located to the periplasm (Figure 9). The hypersensitivity of the *recA* strain, defective in recombinational repair, provides good evidence that DNA breaks are occurring as a result of ROS production. The *mutT* product removes oxidatively damaged nucleotides from the pool so that they cannot be misincorporated into DNA or RNA, providing further evidence for the intracellular ROS production arising from photoactivation. Taken together, the evidence suggests a non-specific mechanism by which Compound **2** activation produces ROS throughout the cell, leading to extensive damage of lipids, proteins and DNA. *E. coli* was able to resume logarithmic growth after an extended lag phase (10 h; Figure 4A), potentially suggesting that outer membrane damage may be repairable. However, it is more likely that a few survivors of the initial photoactivation, recover in its absence and begin to repopulate the culture. However, when Compound **2** enters Gram-positive cells, damage to the cell wall and intracellular components leads to irreparable damage and rapid cell death. It is uncertain whether the breakdown of the cell surface occurs before cell death or is a consequence of cell death. Regardless, Compound **2** has already proven a potent photosensitiser that could prove useful as a light-based antimicrobial treatment for Gram-positive infections, reducing the burden of AMR.

## Data availability statement

The original contributions presented in the study are included in the article/Supplementary material, further inquiries can be directed to the corresponding authors.

## Author contributions

RW, GS, and CAA conceived the study. RW, JH, DC, GS, and CAA wrote the manuscript. RW, CTA, DC, JGH, ED, and EW performed the experiments. All authors contributed to the article and approved the submitted version.

## Conflict of interest

CTA, DC, CS, JH, ED, AW, and CAA were employed by the company LightOx Limited. CAA and AW own shares of LightOx Limited, the company licensed to pursue commercial applications of the novel chemicals described in this manuscript.

The remaining authors declare that the research was conducted in the absence of any commercial or financial relationships that could be construed as a potential conflict of interest.

## Publisher’s note

All claims expressed in this article are solely those of the authors and do not necessarily represent those of their affiliated organizations, or those of the publisher, the editors and the reviewers. Any product that may be evaluated in this article, or claim that may be made by its manufacturer, is not guaranteed or endorsed by the publisher.

## Supplementary material

The Supplementary material for this article can be found online at: https://www.frontiersin.org/articles/10.3389/fmicb.2023.1243818/full#supplementary-material
