# Supplementary material for: The antibacterial activity of a photoactivatable diarylacetylene against Gram-positive bacteria
Source: Front Microbiol. 2023 Sep 22;14:1243818. doi: 10.3389/fmicb.2023.1243818 (PMC10556703; doi:10.3389/fmicb.2023.1243818)
Supplement: Supplementary file 1 [file Table_1.DOCX]

| **Solvent** | **﻿λ_abs_(max)/nm, (ε/M^-1^ cm^-1^)** | **λ_em_(max)/nm** | **φ** | **τ/ns** |
| --- | --- | --- | --- | --- |
| Toluene | 389 | 491 | 0.58 | 1.64 |
| CHCl_3_ | 384 (35700 +/- 1200) | 531 | 0.50 | 2.33 |
| DMSO | 396 (28400 +/- 500) | 588 | 0.0013 | - |
