# Supplementary material for: The antibacterial activity of a photoactivatable diarylacetylene against Gram-positive bacteria
Source: Front Microbiol. 2023 Sep 22;14:1243818. doi: 10.3389/fmicb.2023.1243818 (PMC10556703; doi:10.3389/fmicb.2023.1243818)
Supplement: Supplementary file 3 [file Data_Sheet_1.PDF]

Electronic supporting information

The antibacterial activity of a photoactivatable diarylacetylene against Gram-positive bacteria

Ryan Waite[1], Candace Adams[2], David R. Chisholm[2], Cole Sims[2], Joshua G Hughes[1,2,3], Eva Dias[2], Emily White[1], Kathryn Welsby[4], Stanley W. Botchway[4], Andrew Whiting[2], *Gary J. Sharples[1], and *Carrie A. Ambler[1,2]

1. Department of Biosciences, Durham University, Science Site, Durham, DH1 3LE, United Kingdom
2. LightOx Limited, 65 Westgate Road, Newcastle, NE1 1SG, United Kingdom
3. Department of Physics, Durham University, Science Site, Durham, DH1 3LE, United Kingdom
4. Central Laser Facility, Research Complex at Harwell, Science and Technology Facilities Council, Rutherford Appleton Laboratory, Harwell, Didcot, Oxford OX11 0QX, UK

1. Candidate structures for screening experiments 2

2. Compound Synthesis 2

2.1 General chemical information 2

2.2 5-Iodothiophene-2-carbaldehyde, 7 2

2.3 5-[2-(Trimethylsilyl)ethynyl]thiophene-2-carbaldehyde, 8 3

2.4 Tert-butyl (2E)-3-{5-[2-(trimethylsilyl)ethynyl]thiophen-2-yl}prop-2-enoate, 9 3

2.5 Tert-butyl (2E)-3-(5-ethynylthiophen-2-yl)prop-2-enoate, 10 3

2.6 Tert-butyl (2E)-3-(5-{2-[4-(piperazin-1-yl)phenyl]ethynyl}thiophen-2-yl)prop-2-enoate, 2 3

3. ^{1}H and ^{13}C NMR Spectra 4

4. Photophysical characterisation of compound 2 9

5. Non-irradiated and DMSO controls of compound screening 9

6. Ethanol controls of Propidium Iodide assays 10

7. Time lapse of membrane integrity following photoactivation 11

8. References 11

1. Candidate structures for screening experiments

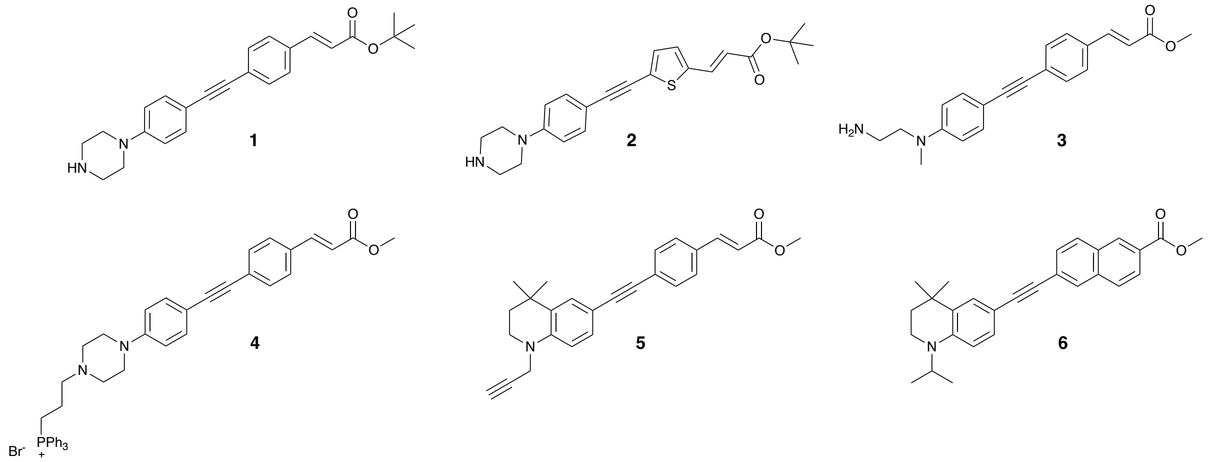

Figure S1: Chemical structures of the six candidate screening compounds.

2. Compound Synthesis

2.1 General chemical information

Reagents were purchased from Sigma-Aldrich, Acros Organics, Alfa-Aesar and Fluorochem. They were purified, if required, by recrystallisation or distillation/sublimation under vacuum. Solvents were used as supplied from Fisher Scientific or Sigma Aldrich, and dried before use if required with appropriate drying agents. Thin-layer chromatography (TLC) was conducted using Merck Millipore silica gel 60G F254 25 glass plates and/or TLC-PET foils of aluminium oxide with fluorescent indicator 254 nm (40 × 80 mm) with visualisation by UV lamp or appropriate staining agents. Flash column chromatography was performed using SiO_2 from Sigma-Aldrich (230-400 mesh, 40-63 µM, 60 Å), and monitored using TLC. NMR spectra were recorded using Bruker Avance Neo-700, Bruker Avance-600 or Bruker Avance-400 spectrometers operating at ambient probe temperature. NMR peaks are reported as singlet (s), doublet (d), triplet (t), quartet (q), broad (br), septet (sept), combinations thereof, or as a multiplet (m), with reference to the following deuterated solvent signals: $CDCl_3$ (^{1}H = 7.26 ppm, ^{13}C = 77.0 ppm), $(CD_3)_2SO$ (^{1}H = 2.50 ppm, ^{13}C = 39.5 ppm). ESMS was performed using a TQD (Waters Ltd., UK) mass spectrometer with an Acquity UPLC (Waters Ltd., UK), and accurate mass measurements were obtained using a QtoF Premier mass spectrometer with an Acquity UPLC (Waters Ltd., UK). IR spectra were recorded using a Perkin Elmer FTIR spectrometer.

2.2 5 - Iodothiophene - 2 - carbaldehyde, 7

To a solution of 2-thiophenecarboxaldehyde (37.4 mL, 400.0 mmol) in EtOH (300 mL) at 50 °C was added *N*-iodosuccinimide (99.0 g, 440.0 mmol) and *p*-toluenesulfonic acid monohydrate (7.60 g, 40.0 mmol), whereupon the resultant solution was stirred at 50 °C for 50 min. The solution was cooled to 35 °C whereupon 1M HCl (500 mL) was added, and the mixture was then extracted with EtOAc, washed with sat. $Na_2S_2O_3$, sat. $NaHCO_3$, H_2O and brine, dried ($MgSO_4$) and evaporated to give compound **7** as a brown oil that slowly crystallised (85.2 g, 90%): ^{1}H NMR (400 MHz, $CDCl_3$) δ 7.39 (s, 2H), 9.77 (s, 1H); ^{13}C NMR (101 MHz, $CDCl_3$) δ 87.8, 137.0, 138.2, 149.6, 181.1. All other data matched the literature.[1] Note: the material contained residual succinimide: ^{1}H NMR (400 MHz, $CDCl_3$) δ 2.77 (s, 4H); along with trace EtOAc and EtOH.

2.3 5‐[2‐(Trimethylsilyl)ethynyl]thiophene‐2-carbaldehyde, 8

Compound **7** (78.3 g, 329 mmol) was dissolved in Et_3N (1000 mL) and the resulting solution was degassed by sonication under vacuum, replacing the atmosphere with Ar (10x). Trimethylsilylacetylene (50.1 mL, 362 mmol), $Pd(PPh_3)_2Cl_2$ (2.31 g, 3.29 mmol) and CuI (0.64 g, 3.29 mmol) were then added under Ar and the resultant suspension was stirred at 40 °C for 18 h. The mixture was diluted with Et_2O and passed through Celite/SiO_2 to give a crude brown oil (72 g). This was purified by SiO_2 chromatography (cyclohexane/EtOAc, 9:1) to give compound **8** as a light brown oil that slowly crystallised (64.22 g, 94%): ^{1}H NMR (400 MHz, $CDCl_3$) δ 0.26 (s, 9H), 7.25 (d, *J* = 3.9 Hz, 1H), 7.61 (d, *J* = 3.9 Hz, 1H), 9.84 (s, 1H); ^{13}C NMR (101 MHz, $CDCl_3$) δ -0.5, 26.9, 96.3, 104.6, 132.5, 133.1, 135.7, 143.8, 182.4; IR (ATR) ν_{max}/cm^{-1} 2960w, 2899w, 2833w, 2148m, 1666s, 1438s, 1249s, 1223s, 1207s, 838s; MS (ES) *m/z* = 209.0 $[M+H]^+$; HRMS (ES) calcd. for $C_{10}H_{13}SOSi$ $[M+H]^+$: 209.0451, found 209.0454.

2.4 *Tert*‐butyl (2*E*)‐3‐{5‐[2‐(trimethylsilyl)ethynyl]thiophen‐2‐yl}prop‐2‐enoate, 9

Tert-butyl diethylphosphonoacetate (40.6 mL, 173 mmol) and LiCl (7.33 g, 173 mmol) were added to anhydrous THF (700 mL) at 0 °C and the resultant solution was stirred for 15 min, whereupon compound **8** (30.0 g, 144 mmol) was added. To this solution was slowly added DBU (25.9 mL, 173 mmol), and the resultant slurry was stirred at RT for 16 h. This was poured into crushed ice and extracted with EtOAc. The organics were washed with H_2O and brine, dried ($MgSO_4$) and evaporated to give a crude brown oil (49.6 g). This was purified by SiO_2 chromatography to give compound **9** as an orange oil, containing approximately 25% of the TMS-deprotection product, compound **10** (37.47 g, 85%): ^{1}H NMR (400 MHz, $CDCl_3$) δ 0.25 (s, 8H), 1.51 (s, 9H), 6.13 (dd, *J* = 15.7, 0.4 Hz, 1H), 7.05 (d, *J* = 3.9 Hz, 1H), 7.12 (d, *J* = 3.9 Hz, 1H), 7.57 (dd, *J* = 15.7, 0.4 Hz, 1H). Note: the percentage yield of this step was calculated on the basis of compound **9**; TMS deprotection was observed during purification on SiO_2.

2.5 *Tert*‐butyl (2*E*)‐3‐(5‐ethynylthiophen‐2‐yl)prop‐2‐enoate, 10

Compound **9** (37.47 g, 122 mmol) was dissolved in a mixture of DCM (300 mL) and MeOH (30 mL), whereupon K_2CO_3 (33.7 g, 244 mmol) was added. The resultant suspension was stirred at RT for 18 h before being diluted with DCM and H_2O. The organics were washed with sat. NH_4Cl and H_2O, dried ($MgSO_4$) and evaporated to give a crude orange oil (28 g). This was purified by SiO_2 chromatography (95:5, cyclohexane/EtOAc) to give compound **10** as an orange oil that slowly darkens (26.33 g, 83%): ^{1}H NMR (300 MHz, $CDCl_3$) δ 1.51 (s, 9H), 3.44 (s, 1H), 6.15 (d, *J* = 15.7 Hz, 1H), 7.07 (d, *J* = 3.8 Hz, 1H), 7.17 (d, *J* = 3.8 Hz, 1H), 7.58 (dd, *J* = 15.7, 0.6 Hz, 1H); ^{13}C NMR (101 MHz, $CDCl_3$) δ 26.9, 28.1, 80.8, 83.4, 120.2, 124.0, 130.0, 133.8, 135.1, 141.2, 165.7; IR (ATR) ν_{max}/cm^{-1} 2978w, 2933w, 1702m, 1623m, 1447m, 1392w, 1368m, 1148s, 753m; MS (ES) *m/z* = 235.1 $[M+H]^+$; HRMS (ES) calcd. for $C_{13}H_{15}SO_2$ $[M+H]^+$: 235.0787, found 235.0786. Note: Compound 10 was generated and reacted in the next step as soon as possible. Slow polymerisation and darkening occurred if left to stand at RT after 24 h under Ar.

2.6 *Tert*‐butyl (2*E*)‐3‐(5‐{2‐[4‐(piperazin‐1‐yl)phenyl]ethynyl}thiophen‐2‐yl)prop‐2‐enoate, 2

Compound **10** (26.33 g, 112.4 mmol) was dissolved in Et_3N (750 mL) and the solution was degassed by sparging with Ar for 1 h. 1-(4-Iodophenyl)piperazine,[2] compound **11** (29.45 g, 102.2 mmol), $Pd(PPh_3)_2Cl_2$ (3.59 g, 5.11 mmol) and CuI (0.97 g, 5.11 mmol) were then added under Ar and the resultant suspension was stirred at 60 °C for 72 h. The resultant suspension was diluted with DCM and washed with sat. $NaHCO_3$ and water, dried ($MgSO_4$) and evaporated to give a crude orange solid (57 g). This was purified by SiO_2 chromatography (95:5, DCM/MeOH, 1% Et_3N) followed by recrystallisation from MeCN to give compound **2** as a yellow/orange solid (27.01 g, 67%): ^{1}H NMR (400 MHz, $CDCl_3$) δ 1.51 (s, 9H), 2.98 – 3.05 (m, 4H), 3.17 – 3.25 (m, 4H), 6.12 (d, *J* = 15.7 Hz, 1H), 6.80 – 6.88 (m, 2H), 7.08 (d, *J* = 3.9 Hz, 1H), 7.10 (d, *J* = 3.8 Hz, 1H), 7.36 – 7.43 (m, 2H), 7.59 (dd, *J* = 15.7, 0.6 Hz, 1H); ^{13}C NMR (101 MHz, $CDCl_3$) δ 28.1, 45.9, 49.1, 80.6, 81.1, 96.4, 112.0, 114.8, 119.2, 126.4, 130.6, 131.8, 132.6, 135.5, 140.1, 151.6, 165.9; IR (ATR) ν_{max}/cm^{-1} 2977w, 2929w, 2820w, 2194w, 1698s, 1617m, 1602m, 1526w, 1323m, 1141s, 812w; MS(ES): *m/z* = 395.3 $[M+H]^+$; HRMS (ES) calcd. for $C_{23}H_{27}N_2O_2S$ $[M+H]^+$: 395.1793, found 395.1792.

3. ^{1}H and ^{13}C NMR Spectra

5-Iodothiophene-2-carbaldehyde, 7

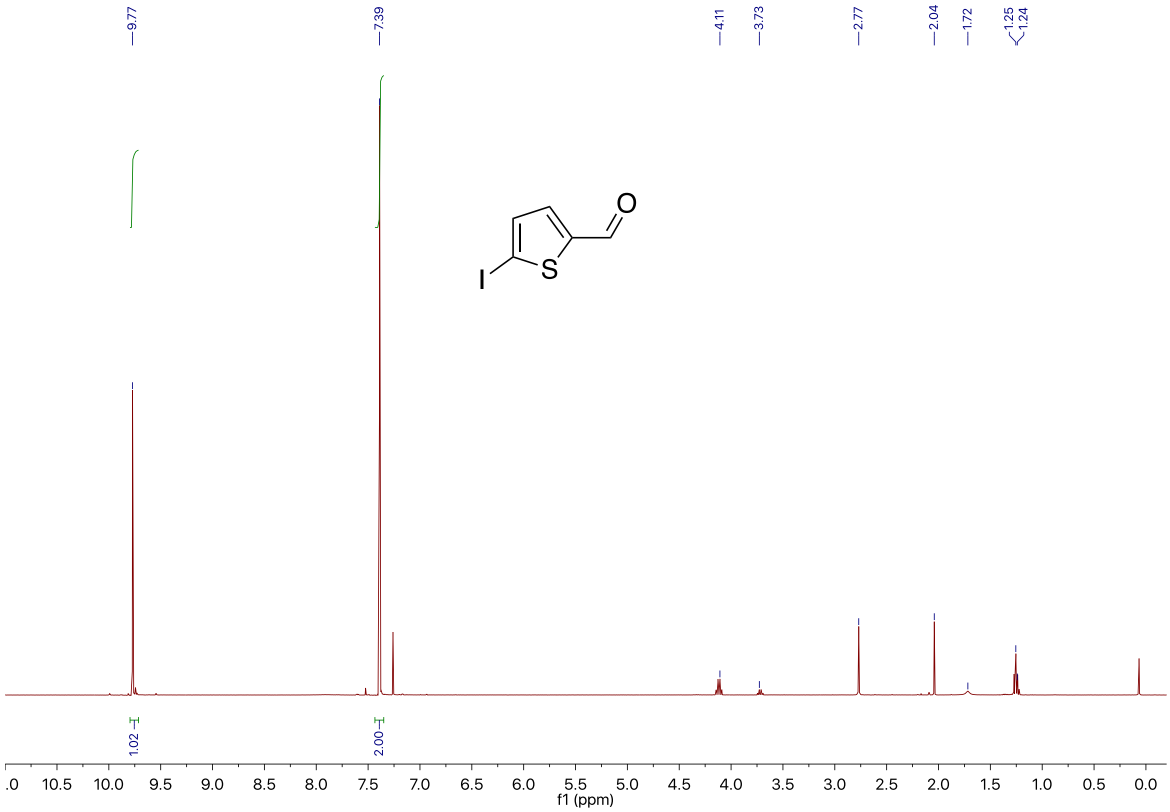

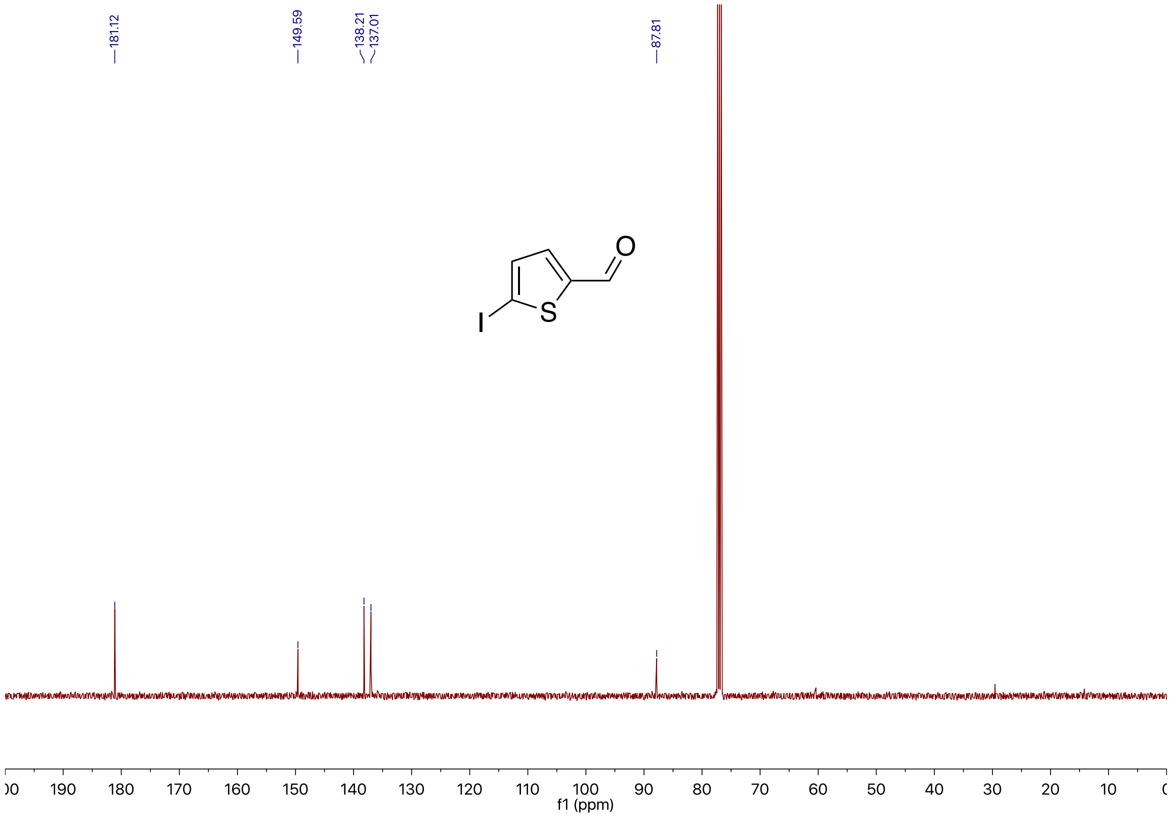

5-[2-(Trimethylsilyl)ethynyl]thiophene-2carbaldehyde, 8

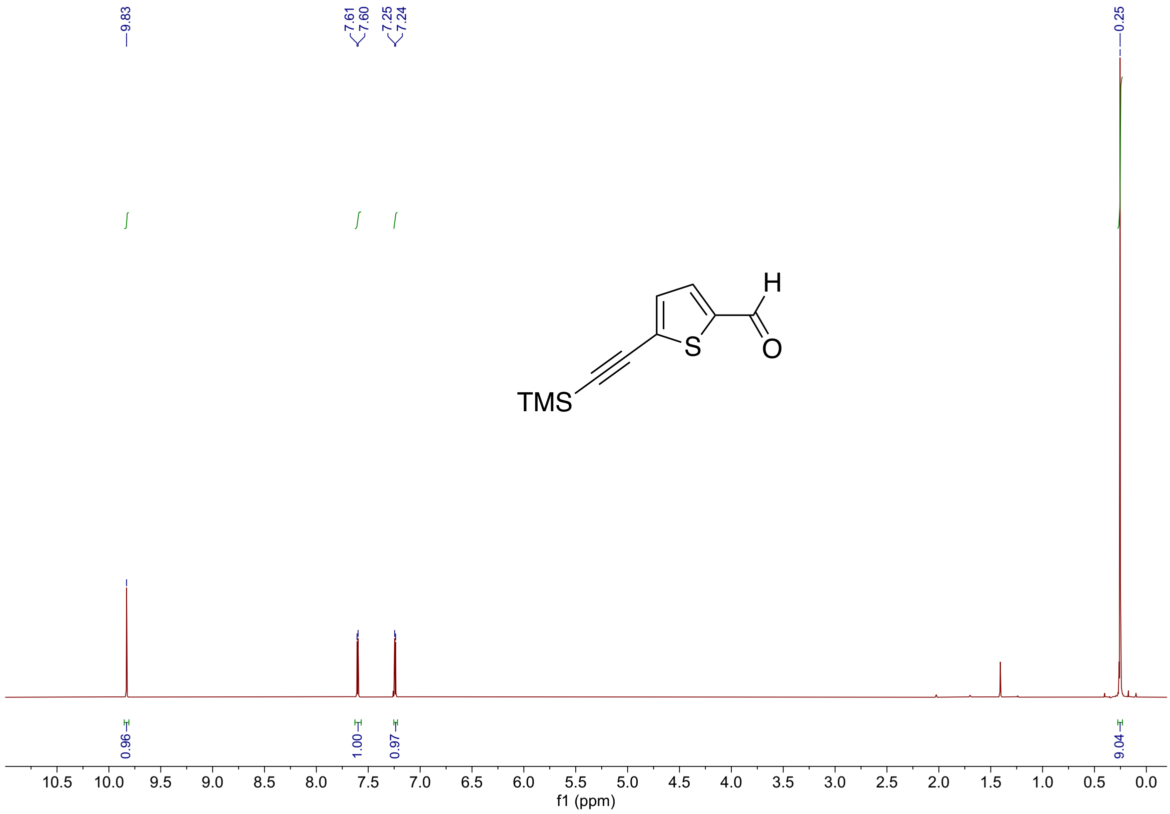

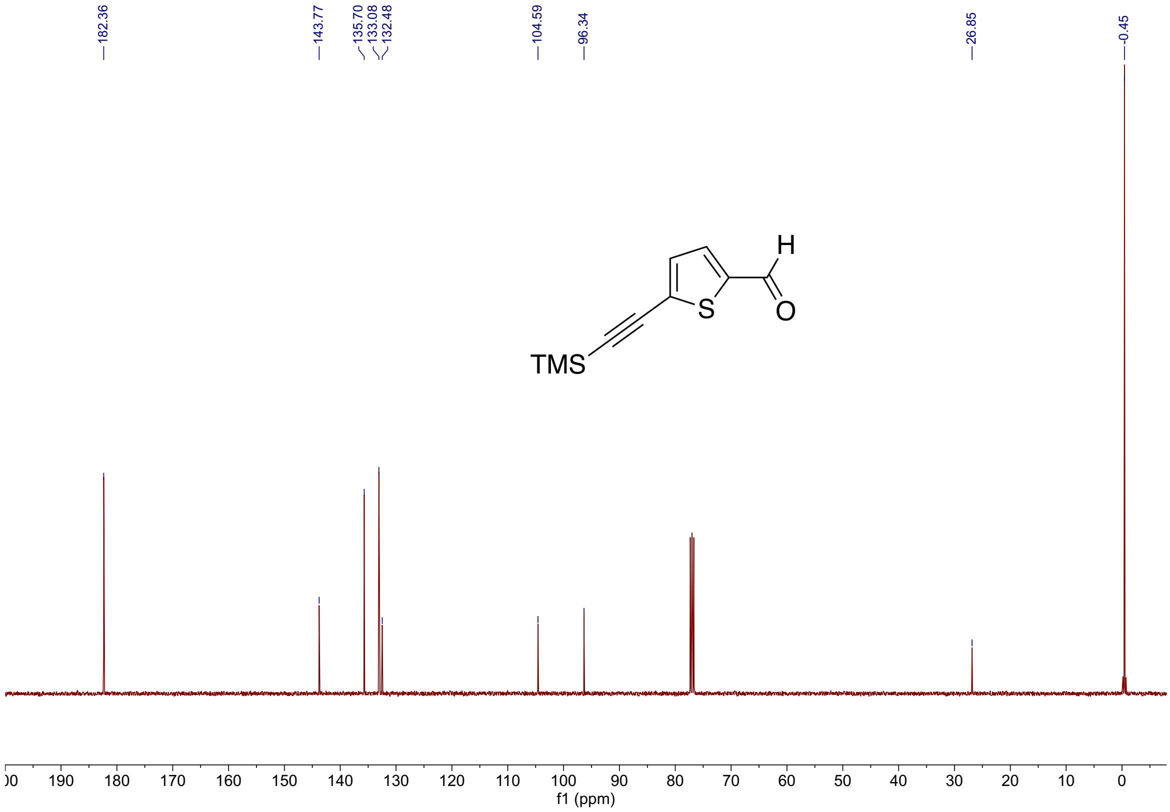

Tert-butyl (2_E_)-3-{5-[2-(trimethylsilyl)ethynyl]thiophen-2-yl}prop-2-enoate, 9

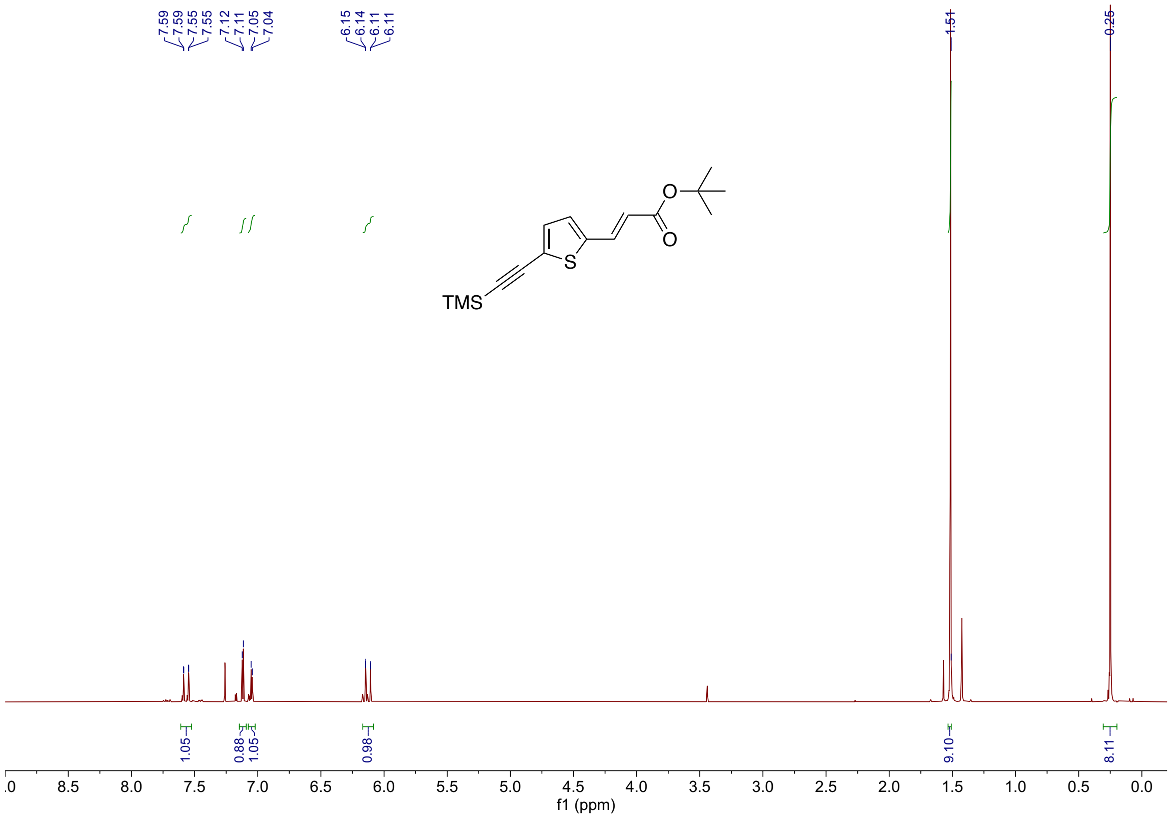

Tert-butyl (2_E_)-3-(5-ethynylthiophen-2-yl)prop-2-enoate, 10

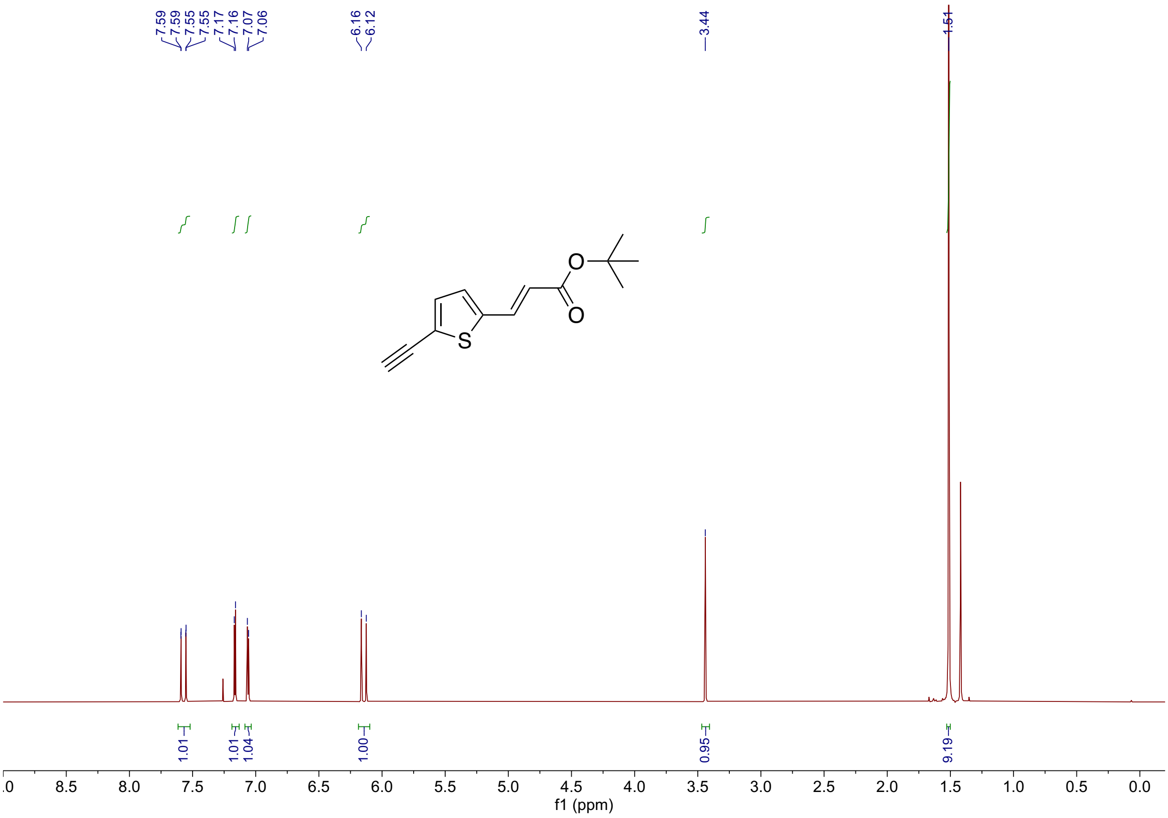

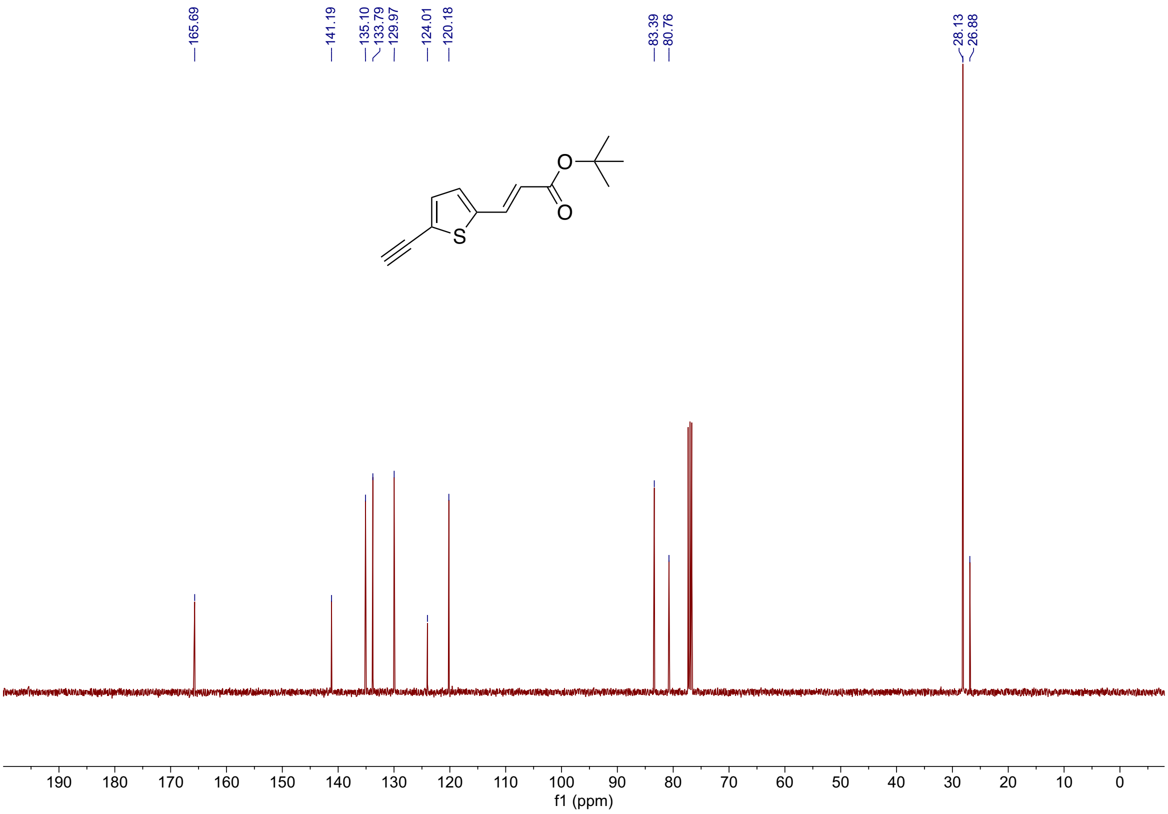

***Tert*-butyl (2*E*)-3-(5-{2-[4-(piperazin-1-yl)phenyl]ethynyl}thiophen-2-yl)prop-2-enoate, 2**

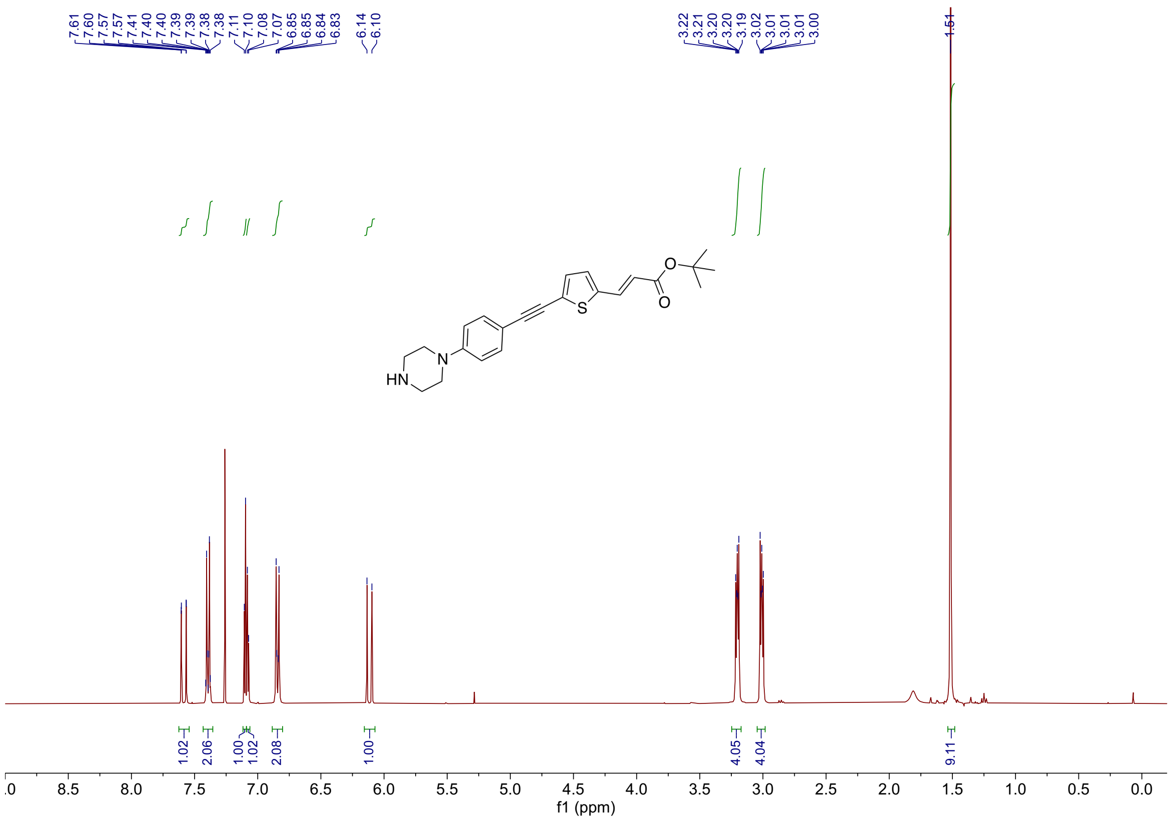

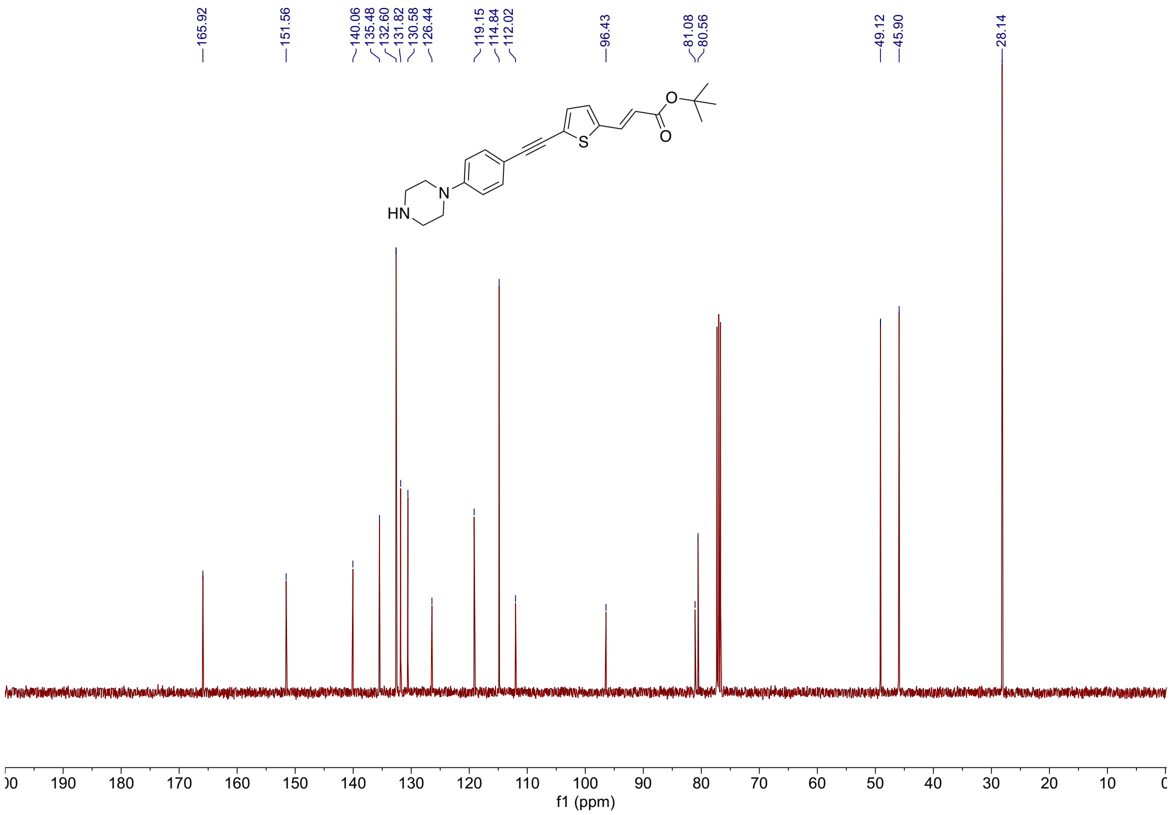

4. Photophysical characterisation of compound 2

Solvent	λ_{abs}(max)/nm, (ε/M^{-1} cm^{-1})	λ_{em}(max)/nm	φ	τ/ns
Toluene	389	491	0.58	1.64
$CHCl_3$	384 (35700 +/- 1200)	531	0.50	2.33
DMSO	396 (28400 +/- 500)	588	0.0013	-

Table S1: Photophysical properties of compound 2 in toluene, chloroform and DMSO.

5. Non-irradiated and DMSO controls of compound screening

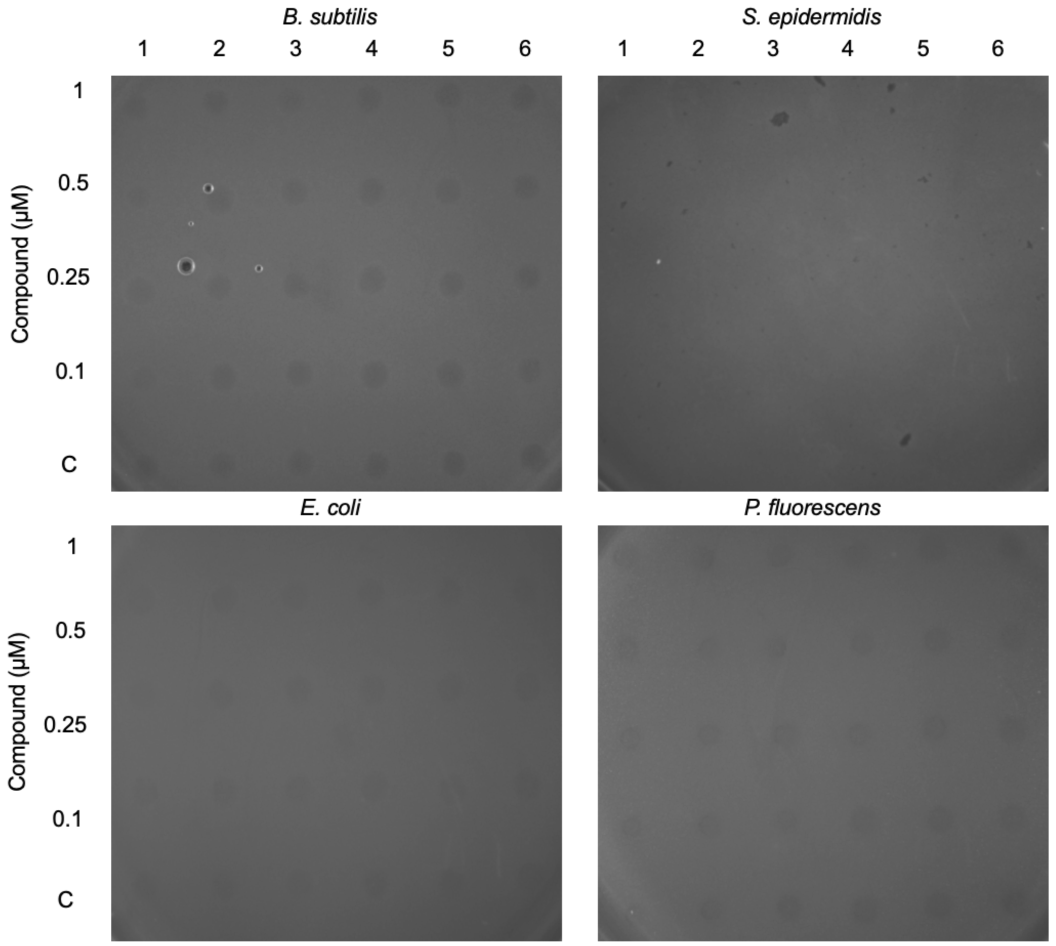

Figure S2. Controls for screening lead compounds for antibacterial activity against Gram-positive and Gram-negative bacteria. Two-fold dilutions of six lead compounds were applied in 6 µl volumes to the surface of a soft agar overlay inoculated with *B. subtilis*, *S. epidermidis*, *E. coli* or *P. fluorescens*. With a control of 0.1% DMSO (C) spotted onto the bottom row. The LB agar plates were incubated for 24 hours at 37°C prior to imaging. Minimal zones of inhibition were seen relative to the photoactivated plates. All spots appear to leave a slight zone of growth inhibition regardless of compound concentration, suggesting the simple application of liquid to the surface of the overlay affects the normal growth of the bacterial lawn beneath.

6. Ethanol controls of Propidium Iodide assays

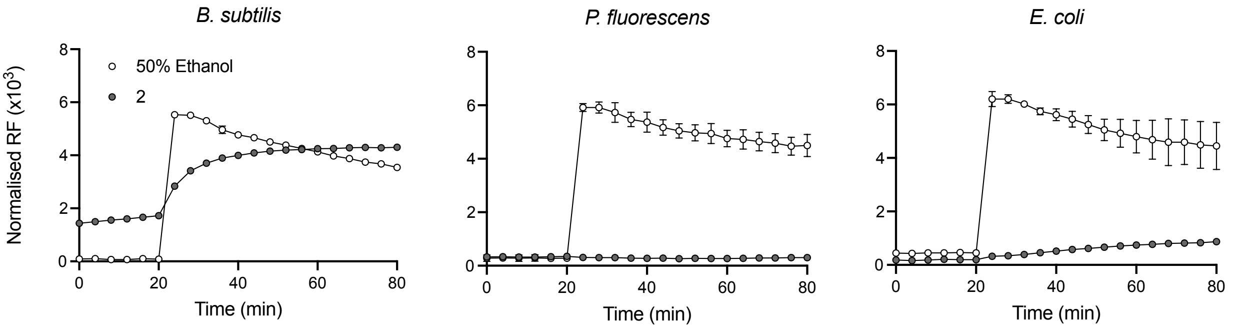

Figure S3. Effect of compound 2 and 50% ethanol on bacterial membrane integrity. Bacteria were grown to mid-log phase in the presence or absence of 2 µM compound 2 as described in the Material and methods. When samples containing compound 2 were photoactivated 50µl of 100% ethanol was added to 50µl of bacteria suspended in PBS to give a final concentration of 50% ethanol. The relative fluorescence units (RFU) at an emission of 645 nm were normalised against controls containing appropriate control concentrations of DMSO.

7. Time lapse of membrane integrity following photoactivation

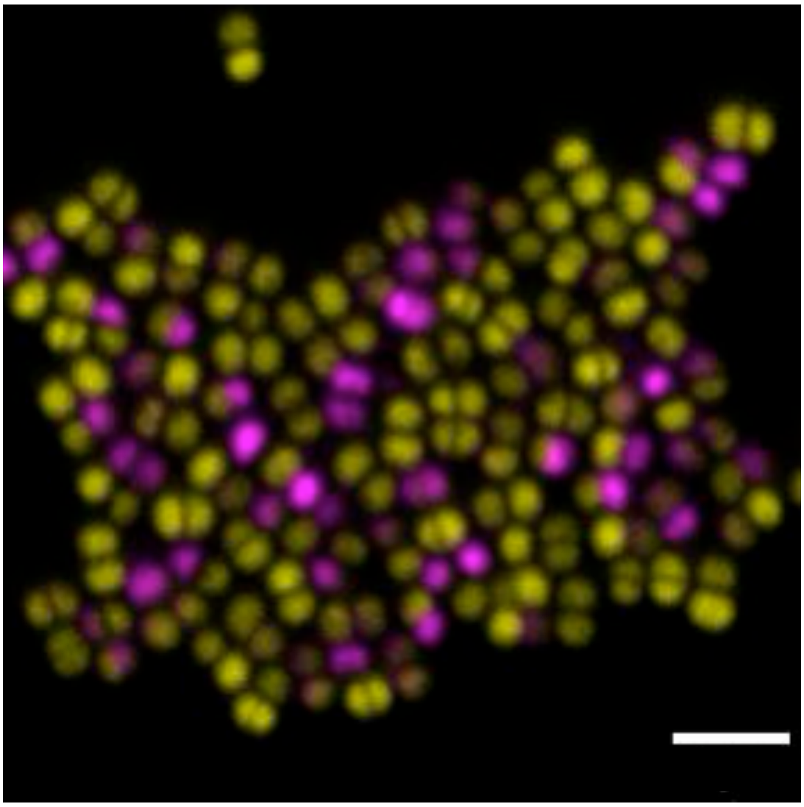

Supplementary Video 1. Real-time monitoring of *S. epidermidis* membrane integrity. The BacLight assay of membrane integrity following photoactivation of compound 2. Bacteria in the mid-log phase of growth were stained with PI (magenta) and SYTO 9 (yellow) and imaged by confocal microscopy using a time series function, with images taken every 6 seconds for 10 mins. Showing the gradual colour change as the PI displaces the SYTO9 within the cell. Scale bar is at 3μM. The laser was applied at 30% power for 1 minute.

8. References

1. J. Grolleau, P. Frère and F. Gohier, *Synthesis (Stuttg).*, 2015, **27**, 3901–3906.

2. D. R. Chisholm, J. G. Hughes, T. S. Blacker, R. Humann, C. Adams, D. Callaghan, A. Pujol, N. K. Lembicz, A. J. Bain, J. M. Girkin, C. A. Ambler and A. Whiting, *Org. Biomol. Chem.*, 2020, **18**, 9231–9245.
