# Supplementary figures and images for: The antibacterial activity of a photoactivatable diarylacetylene against Gram-positive bacteria
Source: Front Microbiol. 2023 Sep 22;14:1243818. doi: 10.3389/fmicb.2023.1243818 (PMC10556703; doi:10.3389/fmicb.2023.1243818)

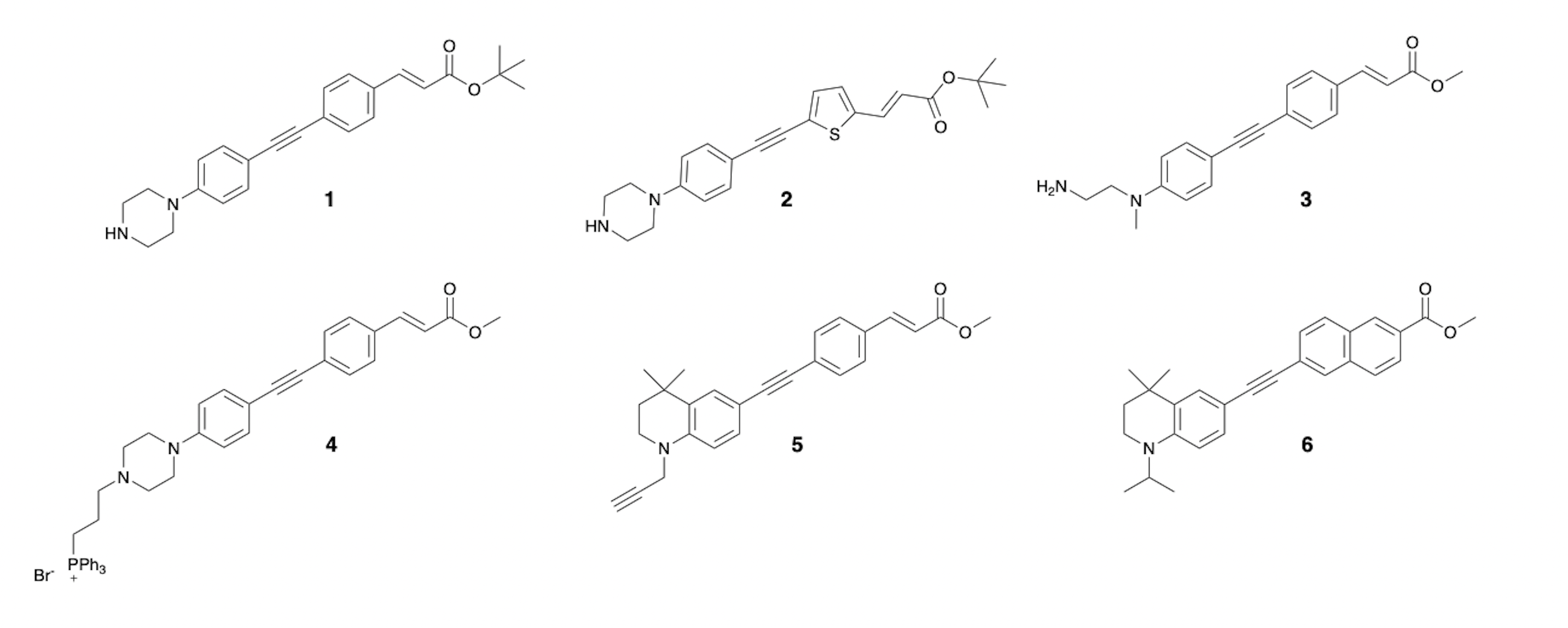

Supplement: Supplementary file 4 [file Image_1.TIFF]

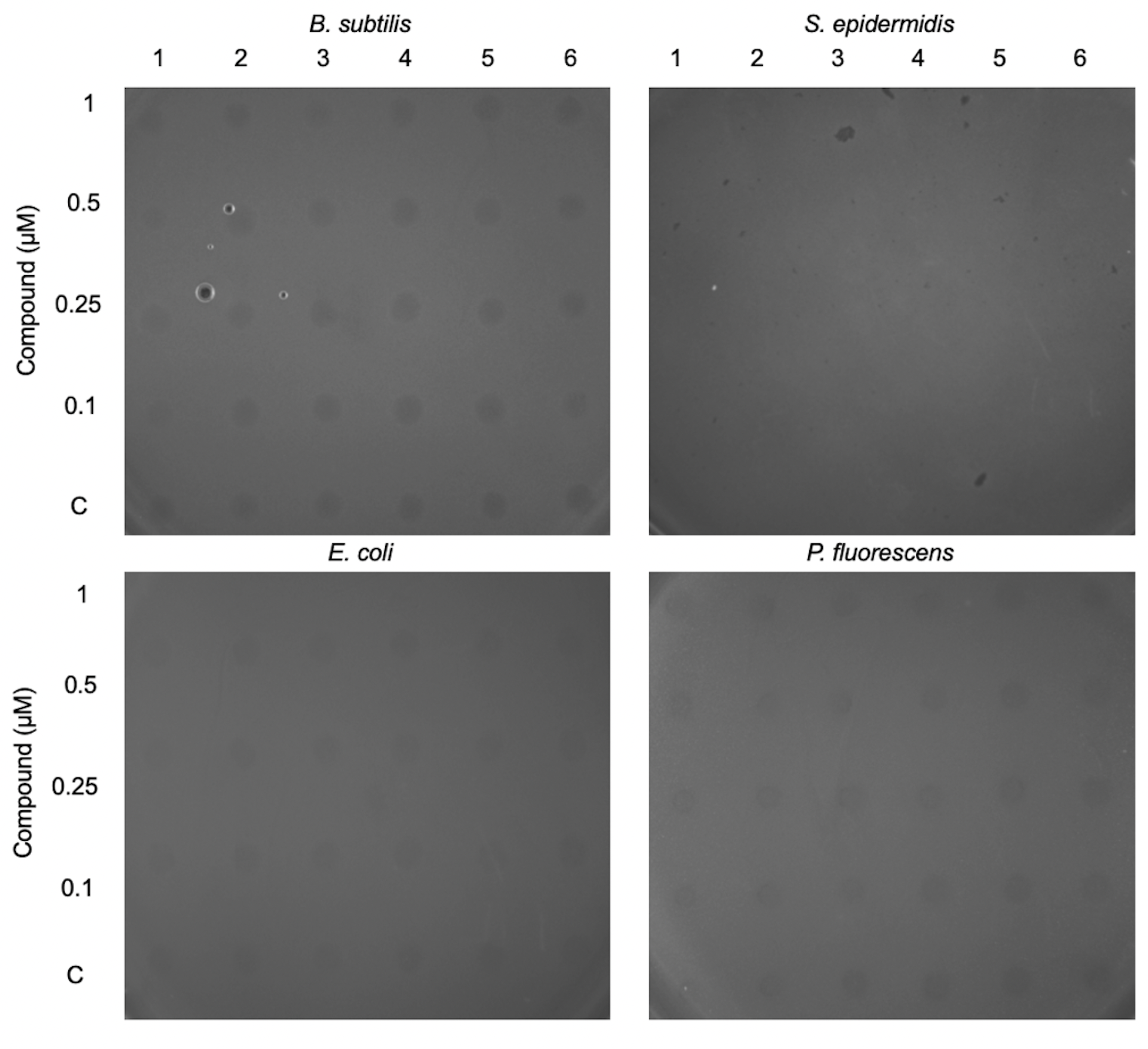

Supplement: Supplementary file 5 [file Image_2.TIFF]

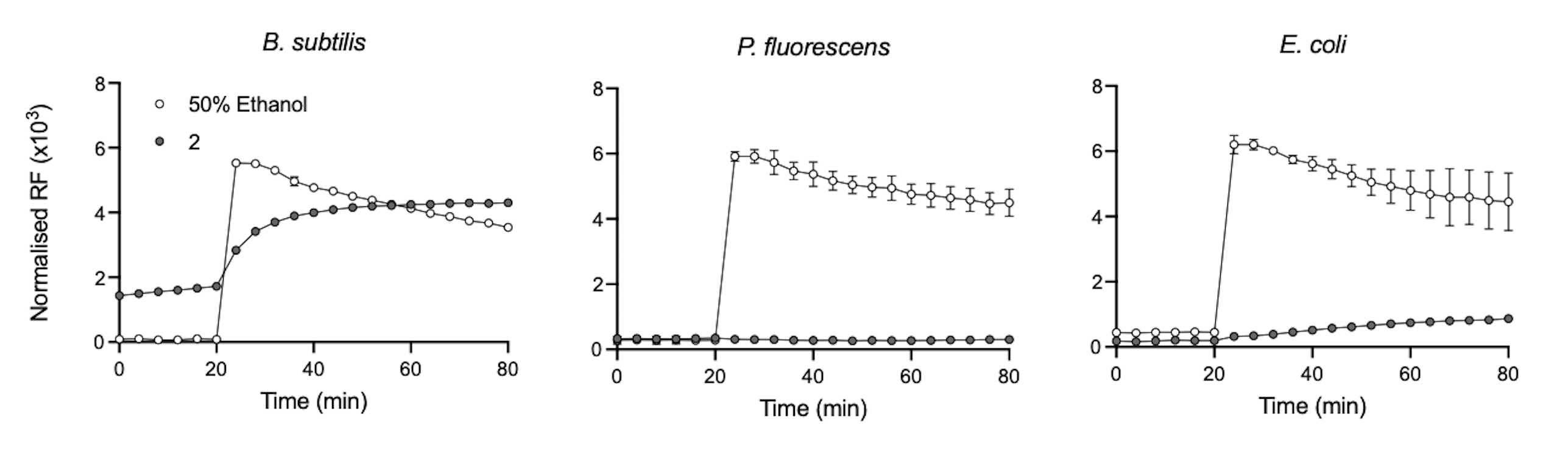

Supplement: Supplementary file 6 [file Image_3.TIFF]
